# Health inequalities in post-conflict settings: A systematic review

**DOI:** 10.1371/journal.pone.0265038

**Published:** 2022-03-14

**Authors:** Dieudonne Bwirire, Rik Crutzen, Edmond Ntabe Namegabe, Rianne Letschert, Nanne de Vries

**Affiliations:** 1 Faculty of Health, Medicine and Life Science, Department of Health Promotion, CAPHRI Care and Public Health Research Institute, Maastricht University, Maastricht, The Netherlands; 2 Faculté de Santé et Développement Communautaires, Université Libre des Pays des Grands Lacs (ULPGL), Goma, Democratic Republic of the Congo; 3 Maastricht University, Maastricht, The Netherlands; Universitat Wien, AUSTRIA

## Abstract

Conflict can be a primary driver of health inequalities, but its impact on the distribution of social determinants of health is not very well documented. Also, there is limited evidence on the most suitable approaches aiming at addressing health inequalities in post-conflict settings. Thus, we undertook a systematic review of the literature concerning the current knowledge and knowledge gaps about structural determinants of health inequalities and assessed the effects of approaches aimed at addressing health inequalities in post-conflict settings. We performed a systematic search in bibliographic databases such as Web of Science, PubMed, and PsycINFO for relevant publications, as well as institutional websites that are relevant to this topic. The search was initiated in March 2018 and ultimately updated in December 2020. No time or geographical restrictions were applied. The quality of each study included in this review was independently assessed using criteria developed by CASP to assess all study types. *Sixty*-*two articles* were deemed eligible for analysis. The key findings were captured by the most vulnerable population groups, including the civilian population, women, children, internally displaced persons (IDPs), and people with symptoms of mental illness. A considerable range of approaches has been used to address health inequalities in post-conflict settings. These approaches include those used to address structural determinants of health inequalities which are accountable for the association between poverty, education, and health inequalities, the association between human rights and health inequalities, and the association between health inequalities and healthcare utilization patterns. However, these approaches may not be the most applicable in this environment. Given the multifactorial characteristics of health inequalities, it is important to work with the beneficiaries in developing a multi-sector approach and a strategy targeting long-term impacts by decision-makers at various levels. When addressing health inequalities in post-conflict settings, it may be best to combine approaches at different stages of the recovery process.

## Introduction

Promoting health and well-being for all is essential to sustainable development. In recent years, notable progress has been made in this regard, but significant challenges remain as health inequalities persist within and among countries [1]. For instance, it has been reported that in Low and Middle-Income Countries (LMIC), the coverage of reproductive and maternal health services is lower among socioeconomically disadvantaged sub-groups. The proportion of births attended by skilled health care personnel differed (by up to 80%) between the richest and poorest sub-groups within 83 study countries, and the use of modern contraception was at least twice as high among women with secondary schooling or higher than among women with no education in almost half of 71 study countries varying in national socio-economic status [2]. Conflict adversely impacts health and health systems, deteriorating structural and social determinants of health, including economic, social, cultural, environmental, and political systems [3].

Very few empirical studies have measured health equity in conflict-affected settings, but those have suggested that conflict has a significant impact [4]. Most importantly, there is limited evidence on appropriate approaches for health equity in such environments. To accomplish equity, the prerequisite is first to ascertain the presence of inequality so that targeted interventions can be planned, implemented, and monitored to determine the progress [5]. This systematic review aims to collate the current knowledge and identify knowledge gaps about structural determinants of health inequalities within post-conflict settings and assess the effects of approaches aimed at addressing structural determinants of health inequalities in this environment. It contributes to the body of knowledge on structural determinants of health inequalities in post-conflict settings which is also the main outcome of this study.

The social determinants of health in conflict settings reflect and further reinforce these inequalities and the vulnerability of those who are disadvantaged because of poverty, marginalization, and discrimination [6]. Three social determinants are specifically important in conflict settings: (i) the loss of human rights, which can be seen as the first and most important social determinant in a conflict situation; (ii) breaches of medical neutrality, in violation of the Geneva Convention, Article 18; and (iii) progression from stress to distress and disease that results from constant, unremitting exposure to a life-threatening situation [7]. Through its pervasive harmful effects on children, armed conflict is a negative social determinant of child health [8]. Nevertheless, the social determinants affecting children in crisis settings are the same as those affecting children in Low and Middle-Income Countries: (i) poor environment: lack of safe water and sanitation, low-quality housing; (ii) poor nutrition; and (iii) lack of access to health services [7].

There is evidence that conflict can be a primary driver of health inequalities [9]. Specifically, socio-economic status (as measured by education, income or occupation) is a key determinant of health. But, there are other more important determinants of health which are specific to the post-conflict environment [10], such as: 1) differential exposure—which may vary (by type, amount, and duration) between social groups 2) differential vulnerability—even when a given risk factor is distributed evenly across social groups, its impact on health may be unevenly distributed due to underlying differences between social groups in their vulnerability or susceptibility to that factor and 3) differential consequences—the impact of a certain health event differs depending on an individual’s or family’s socio-economic circumstances or health. Furthermore, life expectancy and mortality trends are familiar ways of measuring health status and monitoring health inequalities. Countries without basic data on mortality (including post-conflict countries), by socioeconomic indicators might have difficulties progressing their health equity agenda. The example of the DRC illustrates the links between conflict and regional inequalities in mortality. It indicates that mortality rate was higher in unstable eastern provinces compared to stable provinces, and suggest that life expectancy was lower in more deprived areas.

Through a series of mortality surveys conducted between 2000 and 2004, the International Rescue Committee (IRC) has documented the humanitarian impact of war and conflict in the DRC. These studies estimated that 3.9 million people had died since 1998, arguably making the DRC’s conflict the world’s deadliest crisis since World War II. Less than 10 percent of all deaths were due to violence, with most deaths attributed to easily preventable and treatable health conditions (indirectly caused by conflict—and resulting in inequitable health) such as malaria, diarrhea, respiratory infections, and malnutrition [11]. Furthermore, deaths are often used as the primary indicator for the effect that conflict has on civilians; indeed, the number of these deaths declined in the DRC following an increase in security [12–14].

## Key concepts

To study health inequalities, it is of importance to clarify the following concepts: social determinants *versus* structural determinants of health; inequalities *versus* inequities in health; conflict *versus* post-conflict settings, effects of conflict on health equity, and right to health. Without clearly understanding those key concepts, no one can develop effective policies for health equity in this environment. This paper helps to define those key concepts that are needed to better understand health inequalities in post-conflict settings, such as:

### Social determinants versus Structural determinants of health

Because the concept of ‘social determinants’ refers simultaneously to both the determinants of health and the determinants of inequalities in health, we will expand our review of social determinants of health to include the terms ‘‘structural determinants of health” as we assess the literature on this topic. In this article, the term "structural determinants of health inequalities" refers specifically to the interplay between the socio-economic-political context, structural mechanisms generating social stratification, and the resulting socio-economic position of individuals. These mechanisms configure social groups’ health opportunities based on their placement within hierarchies of power, prestige, and access to resources [15]. We hypothesize that structural determinants affect the distribution of resources and have the potential to influence health inequalities.

### Health inequalities versus health inequities

Health inequalities and health inequities have often been used interchangeably in academic and policy literature. For this paper, *health inequalities* refer to differences in the distribution of a specific factor (such as health status, income, opportunities) between different population groups, while *health inequities*, on the other hand, are inequalities in which the outcome is unnecessary and avoidable, as well as unjust and unfair [16]. Following clarification of their meanings and underlying values, we will now use both terms interchangeably throughout this paper.

### Conflict versus post-conflict settings

There is no consensus in the literature about the definition of post-conflict settings. From a global perspective, post-conflict has been defined as the aftermath of a situation that poses challenges severe enough to threaten the continued existence of an established political, economic, and social system. Post-conflict rarely means that violence and strife have ceased at a given moment in all corners of a country’s territory [17]. For this systematic review, post-conflict countries have been defined as having four characteristics: (i) the signing of a formal peace agreement; (ii) a process of political transition by-elections, military or civilian coups; (iii) increased levels of security; and (iv) a perception among national and international actors that there is an opportunity for peace and recovery [18]. Importantly, not all of these characteristics must be present simultaneously. Post-conflict countries can be volatile. Some countries, such as Angola, Liberia, and the Democratic Republic of Congo (DRC), repeatedly cycle between peace and war [18]. As regards the term ‘conflict-affected’: we use this term because, obviously, the interest in post-conflict areas arises from the very fact that the conflict-affected the health situation of the population.

### Right to health

Good health and well-being are considered human rights. Often, the right to health is associated with access to health care and the construction of health infrastructures. However, the right to health extends further; it should set the conditions for a right to health care systems, facilities, services, and goods (including the underlying determinants of health), which should be sufficiently available, physically and economically accessible, acceptable, and of good quality [19]. In recent years, increasing attention has been paid to good health and well-being by human rights treaty monitoring bodies included in various international instruments adopted under the auspices of the United Nations [20,21] or the World Health Organization [22]. Also, in the more recently acclaimed Sustainable Development Agenda, ambitions relating to health and well-being have been included, aiming to offer a new chance to ensure that everyone can access the highest health and health care standards, not just the wealthiest [1]. Nevertheless, failings and shortcomings affecting proper healthcare caused by, among others, armed conflict, widespread crime, persecution, corruption are daily realities. In contrast, several countries have emerged from war and made steady gains on the road to reconstruction, peacebuilding, and development, which led to reduced inequalities, vulnerability, and exclusion [22,23].

### Effects of conflict on health equity

Many studies have investigated the effects of conflict and its aftermath on health and health systems [24–28]. In general, it has been established that conflict harms health indicators [7,29]. However, the effect of conflict on health equity and the distribution of social determinants of health is not well documented. Also, the extent to which the most vulnerable population sub-groups experience health inequalities in post-conflict settings has not been systematically studied. Using existing literature, this paper addresses this gap.

## Materials and methods

This systematic review was conducted according to internationally accepted guidelines; we adhered to the Preferred Reporting Items for Systematic Reviews and Meta-Analysis statement (S1 Checklist. PRISMA) [30] and the PRISMA extension-checklist (PRISMA-E, which enabled an equity-focused systematic review) [31].

The outcome of interest was structural health inequalities and the population of interest consisted of the most vulnerable population groups [32,33] living in post-conflict areas such as the civilian population, women, children, IDPs, and people with symptoms of mental illness—as these are generally affected the most. The majority of the study population sub-groups was identified beforehand. In particular, we would not feel comfortable discussing the most vulnerable sub-groups in this environment without giving proper attention to women. The reasons for this are multifold: 1) traditional networks that form the foundation of women’s social and family roles may be disrupted during the conflict, 2) women may also leave their communities and husbands for other partners to guarantee a source of food for themselves and their families, and 3) women have to assume the role of the primary breadwinners and caretakers of their families—putting them more at risk [34,35]–for instance, the employment opportunities they could fall back on would be low-paying jobs because they may not meet the educational requirements for a better paying job [36].

While applying a stepwise data extraction approach, we found an additional sub-group of interest which consisted of the civilian population and added this afterward to the final sub-group selection. By civilian population, we mean people who are not active members of the security forces of the state, or members of an organized armed militia or opposition group. Government officials (e.g., members of parliament, governors, and councilors) are also excluded as they are instead seen as representatives of the government of a state. This sub-group is made up of individual civilians who do not belong to any of the various sub-groups that are typically recognized as being most vulnerable to inequalities in health.

### Search strategy

The search of the literature was initiated in March 2018 and ultimately updated in December 2020. The search strategy was comprehensive to allow for a wide variety of study designs and interpretations of structural determinants of health inequalities in post-conflict environments to be included.

We used the PROGRESS-Plus as an organizing framework to assess the structural determinants of health inequalities in post-conflict settings. Using PROGRESS-Plus enables the inclusion of a range of factors and circumvents reliance on any single measure to assess inequities, as this is insufficient. The PROGRESS-Plus acronym stands for: Place of residence, Race/ethnicity/culture/language, Occupation, Gender/Sex, Religion, Education, Socio-economic status, and Social capital [37], the factors identified by WHO as relevant stratifiers that can be used to define social groups [38].

We searched the following bibliographic databases: Web of Science (S3 File), PubMed, and PsycINFO for relevant publications, as well as institutional websites that are relevant to this topic, including those of the World Health Organisation, the Health and Fragile States Network, Evidence aid and the 3i.e (International Initiative for Impact Evaluations) database of systematic reviews. References from included studies were checked to identify other relevant studies. Within the bibliographic databases, we searched for published and unpublished articles and abstracts using three sets of key concepts i.e., post-war, inequality, and health [39], in combination with any of the above-mentioned PROGRESS-Plus factors. Depending on each database’s specifications and to increase the sensitivity to retrieve information, MeSH (Medical Subject Headings) and non-MeSH keywords were used as appropriate. No time or geographical restrictions were applied.

The grey literature was collected from various relevant conferences on this topic (e.g., the Post-Conflict Transitions symposium organized by the World Bank). Lastly, we screened relevant public health journals (e.g., Bulletin of the World Health Organisation; PubMed indexed public health journals, etc.). Given a large number of potentially relevant grey literature on the topic, only peer-reviewed reports were included. We finally contacted experts for advice (e.g., the plan to use PRISMA standard in combination with PRISMA-E was discussed with the editor in chief of the Campbell Collaboration Library).

### Study selection

The first author and an information specialist from Maastricht University independently screened the titles and abstracts of all references retrieved by the search strategy to exclude irrelevant results. Potentially relevant studies about structural determinants of health inequalities in post-conflict settings were primarily assessed and reviewed for eligibility based on specified study characteristics. Publications had to meet the following initial criteria: a) post-conflict environment was explicitly described as study setting; b) the most vulnerable sub-groups were considered as study participants, c) assessing and addressing the structural determinants of health inequalities were seen as interventions, d) qualitative or quantitative (or mixed methods) studies were used. To be included in the final review, publications had to meet the following eligibility criteria (S1 File): (i) contain at least one of the three key concepts (post-war, inequality, and health) (S2 File); (ii) include at least one PROGRESS-Plus acronym of interest and, (iii) have all of the above-described study characteristics (a to d). Studies about structural determinants of health inequalities in contexts other than post-conflict environments were excluded (e.g., natural disasters/post-tsunami, terror victims, intimate partner violence). Abstract-only studies, conference abstracts, studies about ongoing conflict settings, and studies on serving or former military personnel were excluded. All other reasons for exclusion have been documented in a PRISMA chart, and available in supporting information (S1 Fig).

The PRISMA flow diagram represents the search strategy and the study selection process. From the initial search in different bibliographic databases, a total of 4,742 potentially relevant studies were identified; 1,743 studies were duplicates and therefore removed. For the remaining 2,999 studies, 1,773 were excluded based on title screening. Of the remaining 1,226 studies, 886 studies were excluded based on abstracts.

After removing duplicates and screening the titles and abstracts, 340 full-text papers were retrieved and read. Of these, 284 studies were excluded, based on diverse reasons specified in the PRISMA flow diagram [S1 Fig], and 55 articles were selected and included in the final review. The above searches were supplemented with a systematic search of the International Initiative for Impact Evaluations (3i.e) database of systematic reviews. We identified 107 potentially relevant studies from which 7 articles were included in the final review.

As shown in S1 Fig, 62 publications about structural determinants of health inequalities in post-conflict settings were identified and included in this review. All retrieved articles were checked for inclusion and exclusion criteria by two reviewers (DB, GF).

### Data extraction and analysis

All identified indexed records concerning this topic were downloaded into EndNote software by the first author and cross-checked by a second reviewer. Discrepancies and disagreements were discussed. A third reviewer mediated when consensus could not be reached.

A stepwise approach for data extraction was adopted as follows: i) filtering by the title and abstracts for relevance, followed by ii) full-text retrieval and review of all potentially relevant publications by a single reviewer (DB) to determine their eligibility for inclusion in this review iii) reference lists of included articles were manually screened to identify additional studies that our strategy could have missed. We also applied the same approach to the grey literature (S1 Fig).

Based on the study objectives, a predesigned data extraction sheet (S1 Table) was used for retrieving information from the selected studies, including author details, topic, study setting, and population, the phenomenon of interest, study design, type of health inequalities studied, evaluation (study outcomes), research type, study results, and study quality. Segments of text describing approaches/interventions to reduce health inequality within the most vulnerable sub-groups were also extracted.

We analyzed relevant information from included papers and synthesized this using different steps, including i) the textual description of the study, which is the process of identification of key findings from each included primary study; ii) the grouping and clustering by organizing the key findings from individual studies into themes and iii) the content analysis for translating data was thoughtfully applied resulting in the generation of analytical themes.

Data was described, analyzed, criticized, summarized by the most vulnerable sub-groups, and reported as part of the evidence in this systematic review.

Due to the heterogeneity of study settings, populations, and interventions (aiming to reduce health inequalities in post-conflict areas) captured in the included publications, it was impossible to conduct a meta-analysis.

### Quality assessment

The quality of the included studies was assessed using the adapted critical appraisal skills program (CASP) checklist developed for systematic reviews [40], consisting of 10 questions that can be answered with "Yes," ‘‘No,” and ‘‘Can’t tell” about the methodological and reporting issues. This original checklist was designed to be used as an educational pedagogic tool, thus does not suggest a scoring system. Therefore, included studies were assigned an overall score of ‘high’, ‘medium’ or ‘low’ based on methodological quality rating of included papers- a summary is presented in S4 Table. Additionally, we assessed the quality of the studies using different areas such as the research question, study design, data collection methods, data analysis, study findings, and ethics [41,42]. Quality was not used as an exclusion criterion.

## Results and discussion

All included articles were published in English. Most of these were published from 2002 onwards, with at least more than one article published each year. On average, 5 articles were published each year in 2008, 2012, 2014, and 2019 (S2 Table). There were peaks in publications in 2013 (10) and 2015 (9).

Among the included publications, 24 were quantitative studies, 22 were qualitative, and 16 were mixed methods design. Of the total of 62 publications included in our final review, 57 studies were conducted in post-conflict settings. There were 6 studies in which the setting was not always clearly defined.

The majority of the studies were based on data collected in sub-Saharan Africa (27/62 total publications; 43%), followed by South Asia (7/62 total publications; 11%)–suggesting that most conflicts have occurred in both geographic regions in the past decade, and the Middle East (6/62 total publications; 9%), and North Africa region (6/62 total publications; 9%). Small numbers of papers emanated from three regions, including Asia Pacific, South America, and Europe—an indicator that recent peace agreements have been reached in these regions. Only one study collected data in the Russia and Eurasia region.

Publications identified by our search varied with regards to population, context, design, outcome measures, or whether the primary study focus was on health, inequality, post-war, or any PROGRESS-Plus factor of interest, making comparisons difficult. To capture this significant heterogeneity, a narrative synthesis approach was adopted.

The methodological quality of each study included in this review was independently assessed using customized criteria developed by CASP to assess all study types. The quality of the studies ranged from high-quality [43–58], medium-quality [59–95], and low-quality [96–103].

Key findings regarding current knowledge and knowledge gaps about structural determinants of health inequalities in post-conflict settings are presented by the most vulnerable population sub-groups, including (i) the civilian population, (ii) women, (iii) children, (iv) IDPs, and (v) the people with symptoms of mental illness. Additionally, key approaches aimed at addressing health inequalities in this setting are presented.

### Structural determinants of health inequalities by the most vulnerable population sub-groups:

#### Civilian population

The social determinants of health such as infrastructure, poverty, housing, education, and discrimination affect the context in which people are born, grow, live, work, and age and their influence on health [58]; they are major contributors to inequalities in health [104]. We found thirty studies [43,45–47,49,51,55–60,63,66,67,70,71,74,76,80,83,86,89,90,93,94,97,100,101,103] examining the multiple interrelated and complex social determinants of health in conflict and post-conflict environments. Most of these studies [45,46,51,56–58,67,89,93,97,100,103] report an association between poverty, education, and health inequality. For instance, Bisogno and Chong [45] identified that the higher the level of education attained, the higher the earnings.

Within the healthcare setting, education may also be linked to self-efficacy. For example, discussing care options with the healthcare provider and insisting on receiving adequate care [89], suggesting that the higher the level of patient’s education, the greater confidence will be expressed, a finding that is consistent with the literature on the subject [105]. Similarly, a cross-sectional survey examining the factors contributing to the resilience of women in the aftermath of Peruvian demonstrated that low levels of education were all associated with lower resilience [88].

Twelve studies [47,49,55,63,80,83,86,90,93,100,101,103] report on healthcare utilization patterns and how a well-functioning and equitable health system can promote health equity in post-conflict. In particular, one cross-sectional study examining health care utilization patterns for mental, behavioral, and emotional problems in Georgia [47], reported that only just over a third of those with a current mental disorder sought any assistance from health services. Despite meeting the criteria for a current mental health disorder, the other persons did not use health services quite simply because they did not report the presence of mental health problems. This suggests that the demand for facility-based services may decrease in environments where there has been political instability regardless of their availability; thus rebuilding trust in the health system may be as important as rebuilding facilities and improving which services are available [90].

Two studies [49,76] describe the presence of an accountability mechanism and community participation as significant program facilitators. The first study assessed community scorecards feasibility in a post-conflict context through the joint engagement of service providers and community members in the design of patient-centered services. It concluded that joint interface meetings facilitated transparent dialogue between the community and service providers resulting in creative and participatory problem-solving mechanisms and mobilization of resources. The second study examined the accountability mechanisms in a community-driven reconstruction program and found that accountability was translated into informal and oral, rather than formal and written, mechanisms. The findings suggest that an adaptation to the local context enhances the working of accountability but restricts its possible impact as it becomes part of local power dynamics in the process.

Need is the primary determinant of healthcare utilization. In post-conflict settings, health services should be available to users in proportion to need rather than social status, and financing for health services should require proportionately fewer contributions from those with the lowest incomes [53]. Furthermore, healthcare utilization is determined by the need for care, by whether people know that they need care, by whether they trust and want to obtain care, and by whether care can be accessed. Other factors (e.g., poverty, geographic area of residence, sex, age, and language) are also important [106]. More evidence on the instrumental role that health systems can play in reducing inequities is available from countries at peace where integrated health systems approaches have been shown to reduce disparities in access to care and improve financial protection from catastrophic health care costs for the poor [107–110].

#### Women

It is widely recognized that armed conflict has a particularly negative impact on women’s lives [111,112]. They suffer disproportionate effects during and after the war, such as inaccessibility of health and education services and sexual violence [113]. As such, “Gender affects exposure to situations which have an impact on health, dictates who has access to health care services and influences its planning and provision [114]—Pg.2”. We found ten studies [52,61,68,69,73,84,88,92,95,99] explicitly reporting on women. Ultimately, there is some available evidence on the links between social determinants of women’s health and health inequalities in post-conflict settings. For example, Hynes [99] point out how women are harmed by war-related disintegration of health, education, and social services and by the loss of essential environmental assets, such as potable water, sanitation, land, and food.

A study investigating whether differences in women’s rights or gender inequalities were associated with stroke mortality at the country-level confirmed a consistent association between women’s rights inequalities and worse health outcomes [73]. The result indicates that gender inequality status is associated with women’s stroke outcomes.

Two studies [61,68] examined gender justice in post-conflict areas. The first study shows that underlying power inequalities and gender ideologies continue to act against changes in gender relations. The findings suggest a link between men’s rigid adherence to gender roles and expectations and the incidence of sexual and gender-based violence. The other study suggests if gender justice is to be improved, it is necessary to place women, their experiences, and their opinions at the center of thinking about transitional justice mechanisms.

Another study assessing inequalities in access to reproductive and maternal health services in Colombia shows that failure to provide these services to females undermines the goal of universal health coverage [84]. This finding suggests a pattern of marginal exclusion and cumulative patterns of inequality in the reproductive and maternal health care service provided to females affected by armed conflict. Elsewhere in Timor-Leste, factors associated with the non-utilization of health services for childbirth in post-conflict were investigated [52]. The authors found that women residing in rural areas were almost three times more likely not to utilize a health facility for childbirth than their urban counterparts. The authors cite as a possible explanation that women living in urban areas have better transportation, economic status, and access to hospital and health services, which enhance their opportunity for facility delivery.

Although a substantial amount of literature has been published on the provision of Sexual and Reproductive Health (SRH) services in post-conflict settings, there is a lot we do not know yet about how to address the problem. To illustrate, a recent systematic review of monitoring and evaluation indicators for SRH demonstrates that rigorous reporting on a core list of indicators is a prerequisite for identifying the changes that a country wants to see concerning SRH and the investments required to save lives [115].

Women and men have different exposures to situations that affect their health and access to health care, they also have differential power to influence decisions regarding the provision of health services. Moreover, women and men have different needs, including unequal access to and control over power, resources, human rights, institutions, and the justice system. According to Sorenson [116], health care and other social facilities remain inadequate for women’s health in the post-conflict period. As such, they face new challenges (e.g. their traditional roles and relationship) and inherit additional responsibilities. The social transformation occurring in the post-conflict context opens up opportunities that should not be missed for women.

Significant differences in sociodemographic and lifestyle characteristics between men and women were documented in northern Uganda in a cross-sectional community survey. The authors found that more women were divorced and widowed than men; underweight was higher among men than women, with elderly and young men being more likely to be underweight [86]. Further studies [117,118] report the same trends, with underweight being more prevalent among men and overweight more prevalent among women. These findings highlight the importance of continuous health and nutritional assessments of all population groups that reflect local social determinants and family structures.

#### Children

Seven studies [48,62,64,78,95,96,102] explored current knowledge and knowledge gaps of social determinants of health and health inequality in children in post-conflict zones. Two studies [48,62] show how children’s well-being was impacted by the scarcity of resources. This included the shortage of electricity, shortage of clean drinking water, disturbed community life, and unsafe streets and playgrounds in the first study. In the second study, the scarcity of resources was characterized by the chronic lack of specialized facilities to address the complex issue of severe malnutrition.

Two other studies [78,96] describe the post-war educational system. The first study discusses post-war education and gender equity in Sierra Leone [78], it shows that post-war educational reconstruction is unlikely to rectify gender inequities that remain well established within mainstream schooling and in the broader social context. The authors argue that the capacity of education to contribute to gender-based change has not been entirely muted. The second study examines the link between schooling, conflict, and gender differentials in these links [96]. The results indicate that boys’ schooling was more negatively affected than that of girls because boys are withdrawn by their families to contribute to the household income or because they are drafted into (armed) serving in the conflict.

In a study of the effects of the conflict on mortality both in utero and during the first year of life in the DRC, Dagnelie and colleagues [64] show that in utero exposure to conflict negatively affects the number of males (by a reduction of the number of male fetuses) reaching birth. The authors show that conflict exposure increases infant mortality specifically among girls. Possible explanations for such an outcome are in utero biological factors. In another study discussing why achieving gender equality is of fundamental importance to improve the health and well-being of future generations, Roseboom [95] notes that prenatal exposure to violence has been associated with long-term effects on children’s health, especially on brain structure and neural function, that can still be detected many years later. A case study examining the role that young boys and girls assume in negotiating household poverty and enhancing their livelihood opportunities in small-scale mining communities from rural sub-Saharan Africa [102] reveals that the implications of their involvement are often complex than international children’s rights advocates understand them to be suggesting that Western notions of “progress” and development often do not match up with the complex realities or competing visions of local people.

Little is known about how the circumstances of a post-conflict setting could affect the health status of children. A recent systematic review of the effects of armed conflict on child health and development [8] shows some gaps in the medium and long-term effects. The authors report limited data on child health and further supports the need for research on how conflict and structural inequalities affect children and subsequent generations. According to the Human Security Centre, child mortality is high in post-conflict countries due to food insecurity leading to malnutrition and the disruption of the water supply and sanitation [119]. This suggests that children are more vulnerable during and after conflicts, as a consequence of a quick transition from conflict to post-conflict in these environments.

#### Internally displaced persons (IDPs)

Most of the time, IDPs are unable to meet their basic needs. They have specific burdens over belonging, housing, occupation, welfare, security, and loss of communities.

Eight studies [44,65,72,75,81,82,85,120] involving IDPs were identified, they collate some evidence of knowledge and gaps about social determinants of health of persons who have been forced to leave their homes due to armed conflict but remained within the borders of their countries. The majority of these studies [65,72,75,81,82] have reported a link between IDPs and poor health outcomes. Specifically, three studies [65,72,81] have reported a long-term effect of conflict on IDP’s health and health care provision. The first study in Bosnia-Herzegovina claims that IDPs remain a forgotten and vulnerable sub-group long after the conflict has ceased and illustrates how the provision and utilization of healthcare services can alleviate some of this vulnerability [72]. The second study investigated the long-term health impacts of internal displacement [65], this study indicated that the IDPs are more likely to report poor self-rated health (SRH) than non-IDPs, suggesting that internally displaced experience poorer health than non-displaced. The third study illustrated medical care among IDPs, in South Sudan [81]. The findings identified that the IDPs have been attracted to services in the hospital because it was representative of improved access to healthcare that could control the health effects associated with displacement and war.

Two studies involving IDPs [44,85] have investigated the relationship between poverty and health inequality in Uganda. The first study explored the relationship between conflict, education, and the intergenerational transmission of poverty. The results confirm that conflict has long-term and intergenerational impacts on well-being and livelihoods [44]. It further confirms that education can limit the poverty impact of conflict on households and support a speedier post-conflict recovery. The other study examined factors that contributed to early relationships and informal marriages in post-conflict settings [85]. The findings note that fundamental shifts in economies and family relationships combined with structural changes encountered in settlements resulted in changed relationships and marriage patterns. In this study, poverty had affected the views and perceptions of the participants around relationships and marriage.

One cross-sectional study of the prevalence of mental disorders in post-conflict primary care attendees (with the majority of participants reporting more than one displacement experience) in the Northern Province of Sri Lanka reports unmet mental health needs in the region and the necessity to address these [120], suggesting that healthcare provision for IDPs remains inadequate and underfunded. This is consistent with previous research findings in northern Uganda where extremely high mortality rates and physical and mental illness amongst IDPs have been recorded [121].

#### People with symptoms of mental illness

We found seven studies [50,54,55,77,79,87,91] examining social determinants of mental health inequalities in post-conflict settings.

Two studies [77,79] assessed the prevalence of mental disorders in post-conflict environments. The first reported a cross-sectional epidemiological study in Nepal where high prevalence rates of mental disorders were found, especially among women, the elderly, widows/widowers, or people separated from their partners. Specifically, depression and anxiety prevalence rates were high compared to other epidemiological studies in high-income settings and comparable to the prevalence rates found in other conflict-affected settings. Study findings underscore the need to address the current lack of mental health care resources in post-conflict rural Nepal, especially for the most vulnerable populations. The second study examined the prevalence and factors associated with Posttraumatic Stress Disorder (PTSD) in northern Uganda. This study found that the prevalence rate of PTSD in the study communities was unacceptably high.

Elsewhere, a study analyzed the relation between exposure to the armed conflict and violence with mental health disorders and assessed the extent and determinants of socioeconomic inequalities in mental health-related to differential exposure to the conflict and violence in Colombia [55]. The findings indicate that victims of armed conflict have a higher probability of suffering mental health disorders compared to non-victims. It shows that differential exposure to armed conflict among lower socio-economic sub-groups explains high proportions of total inequality in mental health disorders.

Two studies [54,87] elaborate on resilience in a post-war context. The first study [54] evaluates a school-based primary prevention intervention designed to promote adolescents’ coping in the immediate aftermath of war exposure. Study findings provide validity for the effectiveness of the intervention in promoting its two targeted resilience factors of mobilization of support and self-efficacy. These findings indicate the need to apply interventions to children at the earliest possible time post-exposure. The second study [87] reports a qualitative, ecological, psychosocial ethnography study in Northern Sri Lanka. It explores psychosocial problems faced by families and communities and the associated risk and protective factors so that practical and effective community-based interventions can be recommended to rebuild strengths, coping strategies, and resilience. Study findings indicate that complex mental health and psychosocial problems at the individual, family, and community levels in a post-conflict context were found to impair recovery.

One qualitative study [50] explored concepts related to mental distress and coping strategies among adults in Burundi. The findings put forward that a general lack of knowledge and information on basic mental health concepts hinders treatment opportunities. For example, most individuals with symptoms of mental illness tend to stay home without receiving any treatment; however, some people seek help in the available infrastructure within their communities [50]. This suggests that more research is needed to know the characteristics of these available infrastructures and how a public mental health model could build from them.

One study exploring the mental health and psychosocial problems of displaced persons living in refugee settings reveals that one consequence of living in situations of pervasive adversity caused by experiences of discrimination, inequity, and violence is poor mental health and psychosocial well-being [91].

Both short- and long-term negative consequences of exposure to the conflict have been documented. Short-term effects included distress, shock, fear, phobic avoidance of public places, anger and emotional pain [122,123], and aggressive behavior [124], while long-term effects included PTSD [125,126], anxiety, and depression [127–129], and sub-clinical symptoms [130]. These findings suggest the need to apply interventions at the earliest possible time post-exposure to traumatic events associated with war and its aftermath affects mental health at both the individual and community levels [54].

### Key approaches aimed at addressing health inequalities in post-conflict settings

We found seventeen publications [46,49,53–56,58,70,80,82,84,89,91,93,97,100,120] assessing approaches aimed to address social determinants of health inequalities in post-conflict settings. The majority of these publications set forth approaches that are believed to be accountable for the association between poverty, education, and health inequalities, such as (i) multi-dimensional local interventions to break the cycles of poverty [46]; (ii) the ‘diseases of the poor’ versus ‘diseases of the rich’ criteria used to prioritize which services to cover [93]; (iii) multi-sectoral approaches [56,58] that pay attention to the political, social and economic determinants of health; and (iv) combined approaches [89,97,100] with different systems being more appropriate at different stages of post-conflict recovery. Notably, two studies [70,100] provide evidence that incorporating economic components into public health interventions is essential for effectiveness across disciplines of global health. Other publications sum up approaches used to address social determinants of health inequalities in relation to healthcare utilization patterns. For instance, (i) interventions that increased quality and access of mental health treatments among victims of the conflict, also improved mental health among victims and significantly reduced inequalities in mental health [55]; (ii) the Community Score Cards (CSC)—a valuable tool for enhancing social accountability for patient-centered care facilitated the flow of information between citizens and service providers [49]; and (iii) a Community Health Workers program increased access to the primary health care services [80].

Other studies outline approaches which are accountable for the association between human rights and health inequalities, such as (i) good governance and respect for human rights reinforce each and influence people’s health [82]; (ii) the Ministry of Health acting as a steward of the health system, by contracting with NGOs to provide services but remaining responsible for the formulation of health policies (e.g., during the development of the Basic Package of Health Services) [53]; (iii) ‘for the people’ drive Community-Based Rehabilitation model (CBR), with equality of rights for people with disabilities (PWD) and their integration into the mainstream of community life [97], and (iv) training of primary care practitioners using a scaled-up intervention based on the WHO Mental Health Gap Action Programme (mhGAP) to address unmet mental health needs [120].

While a range of approaches aimed at addressing health inequalities in stable countries may not be replicable in post-conflict settings, some approaches appear to be promising in a post-conflict environment. For instance, education is essential for instilling stability and normalcy in the lives of children who have experienced war [131]. We found that multi-sectoral approaches and approaches incorporating economic and public health interventions and services prioritization criteria have been successfully used to address this association between poverty, education, and health inequalities.

When women have a role in the decision-making over household economics, the children in the household are healthier and more likely to attend school [132]. Thus, an important opportunity exists to use the microfinance program as a mechanism to provide information to household members on women’s safety and health topics through research [70].

We know now that enhancing community participation can promote positive health practices and motivate the most vulnerable citizens to access health services, better advocate for their needs, and reinforce accountability [133,134]. In Afghanistan, the Community Health Workers (CHW) program has increased access to primary health care services [80]. This finding is consistent with what has been shown in other countries (such as Ethiopia, Iran, Pakistan, and Brazil) with national CHW programs [135–138]. We further found approaches aiming to address the association between human rights and health inequalities. For instance, the Basic Package of Health Services (BPHS) has become a model for the reconstruction of health systems in post-conflict countries. It is a government’s commitment to the population to provide broad coverage of essential services in Afghanistan [139]. This finding suggests that implementation of the BPHS program has resulted in increased access to basic health services for the entire population, especially those in rural areas, thus suggesting improved equity of care [4,140].

Community Driven Reconstruction (CDR) programs have been particularly popular in post-conflict areas. Advocates argue that the model is strong and effective. According to the World Bank, “CDR operations produce two types of results: more and better-distributed assets, and stronger, more responsive institutions—Page 18”. The results from a study of the effects of a large-scale CDR program on the economic well-being and the socio-political attitudes and behaviors of the populations in the DRC were negative [71]. These findings present a challenge to the CDR model and its ability to produce the social and economic impacts that advocates attribute to it [66].

## Study strengths and limitations

Although many studies on health inequalities have been conducted in *stable* contexts, this review is, as far as we know, the first about structural determinants of health inequalities in post-conflict settings. It collates the current knowledge and knowledge gaps and assesses the effects of approaches aimed at addressing structural determinants of health inequalities. Therefore, it adds to a relatively small body of existing literature investigating this topic in a *post-conflict* setting. To identify relevant publications, we conducted a comprehensive and systematic review of the literature. We included quantitative, qualitative, mixed methods and other types of studies in the final review. Of particular interest was the inclusion of studies using at least one PROGRESS-plus factor in the final selection, making this an equity-driven process. There are, however, several limitations to consider when interpreting the findings of the current review.

First, we sought to ascertain the presence of health inequalities so that targeted interventions could be planned, implemented, and monitored. By doing so, we found significant heterogeneity of settings, populations, interventions, and outcomes of interest in the studies that were reviewed, making international comparisons of interventions and outcomes difficult. As such, we used the narrative synthesis to bring together the findings from all included studies; however, it should be noted that this approach is not underpinned by an authoritative body of knowledge or on reliable and rigorous techniques developed and tested over time. In the absence of such a body of knowledge, there is a possibility that systematic reviews adopting a narrative approach to synthesis might be prone to bias. This must be weighed against the counter-argument that we selected our evidence judiciously and purposively with an eye to what is relevant for our main review questions, including any future research questions we would like to address. Instead, the quality of the included studies was assessed systematically.

Second, as with any systematic review, there were concerns about whether we might have missed certain evidence. To be as conclusive as possible, a significant effort was made by applying a rigorous search strategy and identifying articles from scientific and professional databases only.

Third, although we agree and recognize LGBTQ+ (Lesbian, Gay, Bisexual, Trans, Queer, and others) people as belonging to the most vulnerable sub-groups in post-conflict settings—this sub-group was not included in our systematic review because LGBTQ+ issues require special attention. For the same reason, they could not *reasonably* be easily summarized in the current manuscript. To successfully tackle LGBTQ+ issues, special consideration must be given to the particular needs and risks faced by individuals based on their sexual orientation and gender identity. Future systematic reviews on health inequalities within the post-conflict environment should also include LGBTQ+ people as a vulnerable sub-group.

Despite the limitations noted above, some clear themes about structural health inequalities in post-conflict settings were evident. Ultimately, the breadth of the papers and grey literature reviewed, the study selection methods, and the appraisal process altogether provided a substantial body of evidence that supports our key findings reported in the review.

## Conclusion and future directions

This systematic review documents the existing evidence and knowledge gaps about structural health inequalities in post-conflict settings. The results of our review assert the urgent need for addressing identified knowledge gaps as well as the need for developing appropriate approaches aiming at addressing health inequalities in this environment. To shape adequate responses, steps forward should include ways and means to address health inequalities successfully. In particular, priority should be given to studies from the “post-conflict real world”. Such studies should aim to: (1) ensure that the voices of the most vulnerable groups are heard, their active participation as agents of change is guaranteed, and local norms are respected. (2) exploring the novel design of a health system that would ensure both geographic and social equity.

A considerable range of approaches has been used to address health inequalities in post-conflict settings, including those used to address structural determinants that are accountable for the association between poverty, education, and health inequalities, the association between human rights and health inequalities, and the structural determinants of health inequalities in relation to healthcare utilization patterns. However, these approaches may not be the most suitable in this environment. They may not have involved vulnerable groups in decision-making processes and the implementation of strategies targeting relevant drivers of health inequalities.

Given the multifactorial characteristics of health inequalities, it is crucial to work with the beneficiaries in developing a multi-sector approach and a strategy targeting long-term impacts by decision-makers at various levels. Additionally, it may be best to combine approaches aimed to address health inequalities at different stages of the post-conflict recovery process.

This review has strengthened our conviction that there is room for innovation in developing potentially effective “real world” approaches, targetting and involving the most vulnerable groups in addressing health inequalities.

## Supporting information

S1 Checklist PRISMA 2009. (DOC)

S1 Fig PRISMA flow diagram of selection process. (DOC)

S1 Table Data extraction sheet information from selected studies. (XLSX)

S2 Table Characteristics of studies included. (XLSX)

S3 Table Approaches aimed at addressing health inequalities. (XLSX)

S4 Table Individual quality assessment for each study included. (XLSX)

S1 File Eligibility criteria. (DOCX)

S2 File Key concepts and search terms. (DOCX)

S3 File Web of Science search strategy. (DOCX)

## References

[1] United Nations. Good Health and Well-being: Why it matters? [cited 2017. https://www.un.org/sustainabledevelopment/wp-content/uploads/2017/03/ENGLISH_Why_it_Matters_Goal_3_Health.pdf.

[2] World Health Organization, International Center for Equity in Health. State of inequality. Reproductive, maternal, newborn and child health. 2015 [http://apps.who.int/iris/bitstream/10665/164590/1/9789241564908_eng.pdf.

[3] Akseer N, Wright J, Tasic H, Everett K, Scudder E, Amsalu R, et al. Women, children and adolescents in conflict countries: an assessment of inequalities in intervention coverage and survival. BMJ global health. 2020;5(1):e002214. doi: 10.1136/bmjgh-2019-002214 32133179 PMC7042600

[4] Bornemisza O, Ranson MK, Poletti TM, Sondorp E. Promoting health equity in conflict-affected fragile states. Social science & medicine. 2010;70(1):80–8. doi: 10.1016/j.socscimed.2009.09.032 19853342

[5] World Health Organization. Uncovering health inequalities: A path towards leaving no one behind 2016 [http://who.int/features/2016/health-inequalities/en/[Last.

[6] Krug EG, Mercy JA, Dahlberg LL, Zwi AB. The world report on violence and health. Lancet. 2002;360(9339):1083–8. doi: 10.1016/S0140-6736(02)11133-0 12384003

[7] World Health Organization, Regional Office for the Eastern Mediterranean. Social determinants of health in countries in conflict: a perspective from the Eastern Mediterranean Region. World Health Organization; 2008.

[8] Kadir A, Shenoda S, Goldhagen J. Effects of armed conflict on child health and development: A systematic review. PLoS one. 2019;14(1). doi: 10.1371/journal.pone.0210071 30650095 PMC6334973

[9] Coghlan B, Brennan RJ, Ngoy P, Dofara D, Otto B, Clements M, et al. Mortality in the Democratic Republic of Congo: a nationwide survey. Lancet. 2006;367(9504):44–51. doi: 10.1016/S0140-6736(06)67923-3 16399152

[10] Ranson K, Poletti T, Bornemisza O, Sondorp E. Promoting health equity in conflict-affected fragile states. London: The Conflict and Health Programme, London School of Hygiene and Tropical Medicine. 2007. 10.1016/j.socscimed.2009.09.032 19853342

[11] Hale C, Belyakdoumi F, Cobey L, Ondeko R, Despines M. Mortality in Eastern Democratic Republic of Congo-Results form Eleven Mortality Surveys. International Rescue Committee; 2001.

[12] Kandala N-B, Mandungu TP, Mbela K, Nzita KPD, Kalambayi BB, Kayembe KP, et al. Child mortality in the Democratic Republic of Congo: cross-sectional evidence of the effect of geographic location and prolonged conflict from a national household survey. BMC Public Health. 2014;14(1):266. doi: 10.1186/1471-2458-14-266 24649944 PMC4234186

[13] Robinson E, Crispino V, Ouabo A, Soung Iballa FB, Kremer R, Serbassi ME, et al. Mortality and health survey, Walikale, Democratic Republic of the Congo, 2017: an example of the use of survey data for humanitarian program planning. Conflict and Health. 2019;13(1):56. doi: 10.1186/s13031-019-0232-y 31832090 PMC6869257

[14] Rubenstein L. Post-Conflict Health Reconstruction: New Foundations for U.S. Policy. 2007.

[15] World Health Organization. A conceptual framework for action on the social determinants of health. Social Determinants of Health; 2010 13 July 2010. Report No.: 9789241500852.

[16] Whitehead M. The concepts and principles of equity and health. International journal of health services: planning, administration, evaluation. 1992;22(3):429–45. doi: 10.2190/986L-LHQ6-2VTE-YRRN 1644507

[17] Brinkerhoff DW. Governance in Post-Conflict Societies: Rebuilding Fragile States. Brinkerhoff DW, editor. New York, NY: Routledge; 2007.

[18] Macrae J, Zwi A, Forsythe V. Aid policy in transition: a preliminary analysis of “post” conflict rehabilitation of the health sector. Journal of International Development. 1995;7(4):669–84.

[19] Office of the United Nations High Commissioner for Human Rights, World Health Organization. The Right to Health: Fact Sheet 31. Geneva; 2008.

[20] United Nations General Assembly. International covenant on economic, social and cultural rights. Resolution 2200A (XXI) of December. 1966;16:1966.

[21] Baderin M, McCorquodale R. The International Covenant on Economic, Social and Cultural Rights: 40 Years of Development. 2007. doi: 10.1007/s00221-006-0721-2 17031681

[22] World Health Organization. Health in 2015: from MDGs, Millennium Development Goals to SDGs, Sustainable Development Goals. 2015. Report No.: 978 92 4 156511 0.

[23] Ki-Moon B. The road to dignity by 2030: ending poverty, transforming all lives and protecting the planet. Synthesis report of the Secretary-General on the post-2015 sustainable development agenda New York: United Nations. 2014.

[24] Davis D, Kuritsky J. Violent conflict and its impact on health indicators in Sub-Saharan Africa, 1980 to 1997. 2002.

[25] Garfield RM, Neugut AI. Epidemiologic analysis of warfare: a historical review. Jama. 1991;266(5):688–92. 2072480

[26] Ityavyar DA, Ogba LO. Violence, conflict and health in Africa. Social science & medicine (1982). 1989;28(7):649–57. 2711218 10.1016/0277-9536(89)90212-8

[27] Toole MJ, Waldman RJ. The public health aspects of complex emergencies and refugee situations. Annu Rev Public Health. 1997;18(1):283–312. doi: 10.1146/annurev.publhealth.18.1.283 9143721

[28] Zwi A, Ugalde A, Richards P. The effects of war and political violence on health services. Encyclopaedia of violence. 1999:679–90.

[29] Stewart-Brown S. Emotional wellbeing and its relation to health: Physical disease may well result from emotional distress. British Medical Journal Publishing Group; 1998. doi: 10.1136/bmj.317.7173.1608 9848897 PMC1114432

[30] Moher D, Liberati A, Tetzlaff J, Altman DG. Preferred reporting items for systematic reviews and meta-analyses: the PRISMA statement. Annals of internal medicine. 2009;151(4):264–9. doi: 10.7326/0003-4819-151-4-200908180-00135 19622511

[31] Welch V, Petticrew M, Tugwell P, Moher D, O’Neill J, Waters E. PRISMA-Equity 2012 Extension: Reporting Guidelines for Systematic Reviews with a Focus on Health Equity 2012 [Available from: doi: 10.1371/journal.pmed.1001333 23222917 PMC3484052

[32] Brück T, Schindler K. The impact of conflict and fragility on households: A conceptual framework with reference to widows. Research paper/UNU-WIDER; 2008. Report No.: 9292301373.

[33] Haar RJ, Rubenstein LS. Health in postconflict and fragile states: US Institute of Peace; 2012.

[34] Payne L. Impact of food delays on refugees. The Field Exchange, Emergency Nutrition Network. 1997(2):10–1.

[35] Payne L. Rebuilding communities in a refugee settlement: a casebook from Uganda: Oxfam; 1998.

[36] Bodewig C. Emerging from Ethnic Conflict: Challenges for Social Protection Design in Transition Countries. World Bank Institute. 2002:64.

[37] O’Neill J, Tabish H, Welch V, Petticrew M, Pottie K, Clarke M. Applying an equity lens to interventions: Using PROGRESS ensures consideration of socially stratifying factors to illuminate inequities in health. J Clin Epidemiol. 2014:56–64. doi: 10.1016/j.jclinepi.2013.08.005 24189091

[38] World Health Organization. Handbook on health inequality monitoring: with a special focus on low-and middle-income countries. Geneva: World Health Organization,; 2013.

[39] Huber M, Knottnerus JA, Green L, van der Horst H, Jadad AR, Kromhout D, et al. How should we define health? BMJ (Clinical research ed). 2011;343:d4163. doi: 10.1136/bmj.d4163 21791490

[40] CASP, UK. CASP checklists. Oxford Better Value Healthcare Ltd.; 2015.

[41] Coughlan M, Cronin P, Ryan F. Step-by-step guide to critiquing research. Part 1: quantitative research. British journal of nursing. 2007;16(11):658–63. doi: 10.12968/bjon.2007.16.11.23681 17577184

[42] Ryan F, Coughlan M, Cronin P. Step-by-step guide to critiquing research. Part 2: Qualitative research. British journal of nursing. 2007;16(12):738–44. doi: 10.12968/bjon.2007.16.12.23726 17851363

[43] Ayazi TL, Eide L., Swartz A., Hauff L E., Association between exposure to traumatic events and anxiety disorders in a post-conflict setting: a cross-sectional community study in South Sudan. Bmc Psychiatry. 2014;14. 10.1186/1471-244X-14-6 PMC3893536 24410951

[44] Bird K H K. McKay A. Conflict, education and the intergenerational transmission of poverty in Northern Uganda. Journal of International Development. 2010;22(8):1183–96.

[45] Bisogno M C A. On the determinants of inequality in Bosnia and Herzegovina. Econ Transit. 2002;10(2):311–37.

[46] Blandón EZ, Källestål C, Peña R, Perez W, Berglund S, Contreras M, et al. Breaking the cycles of poverty: Strategies, achievements, and lessons learned in Los Cuatro Santos, Nicaragua, 1990–2014. Global Health Action. 2017;10(1):1272884. doi: 10.1080/16549716.2017.1272884 28136698 PMC5328362

[47] Chikovani IM, Gotsadze N, Patel G., McKee V., Uchaneishvili M., Rukhadze M., et al. Health service utilization for mental, behavioural and emotional problems among conflict-affected population in Georgia: a cross-sectional study. PLoS One. 2015;10(4):e0122673. doi: 10.1371/journal.pone.0122673 25853246 PMC4390285

[48] Colombatti RC, Bestagini A., Vieira P., Schiavon C. S., Ambrosini L., Bertinato V., et al. A short-term intervention for the treatment of severe malnutrition in a post-conflict country: results of a survey in Guinea Bissau. Public Health Nutr. 2008;11(12):1357–64. doi: 10.1017/S1368980008003297 18652716

[49] Edward AO-B, Branchini K., Yarghal C., Arwal T. S., Naeem S. H., A J. Enhancing governance and health system accountability for people centered healthcare: an exploratory study of community scorecards in Afghanistan. Bmc Health Services Research. 2015;15. doi: 10.1186/s12913-015-0946-5 26227814 PMC4521484

[50] Familiar IS, Ndayisaba S., Munyentwari H., Sibomana N., Bass S., J K. Community perceptions of mental distress in a post-conflict setting: A qualitative study in Burundi. Global Public Health. 2013;8(8):943–57. doi: 10.1080/17441692.2013.819587 23941217

[51] Finnoff K. Decomposing inequality and poverty in post-war Rwanda: The roles of gender, education, wealth and location. Dev South Afr. 2015;32(2):209–28.

[52] Khanal V L A. H. da Cruz J. L. Karkee R. Factors associated with non-utilisation of health service for childbirth in Timor-Leste: evidence from the 2009–2010 Demographic and Health Survey. BMC Int Health Hum Rights. 2014;14:14. doi: 10.1186/1472-698X-14-14 24885424 PMC4026833

[53] Kruk MEF, L. P. Anglin G. A. Waldman R. J. Rebuilding health systems to improve health and promote statebuilding in post-conflict countries: A theoretical framework and research agenda. Social Science & Medicine. 2010;70(1):89–97. doi: 10.1016/j.socscimed.2009.09.042 19850390

[54] Slone M S A. Lobel T. Helping youth immediately following war exposure: a randomized controlled trial of a school-based intervention program. The journal of primary prevention. 2013;34(5):293–307. doi: 10.1007/s10935-013-0314-3 23868708

[55] Cuartas Ricaurte J, Karim LL, Martínez Botero MA, Hessel P. The invisible wounds of five decades of armed conflict: Inequalities in mental health and their determinants in Colombia. International Journal of Public Health. 2019;64(5):703–11. doi: 10.1007/s00038-019-01248-7 31119303

[56] Sugiura Y, Ju YS, Yasuoka J, Jimba M. Rapid increase in Japanese life expectancy after World War II. Bioscience Trends. 2010;4(1):9–16. 20305339

[57] Guarnizo-Herreño CC, Watt RG, Garzón-Orjuela N, Suárez-Zúñiga E, Tsakos G. Health insurance and education: major contributors to oral health inequalities in Colombia. J Epidemiol Community Health. 2019;73(8):737–44. doi: 10.1136/jech-2018-212049 31097482

[58] Kienzler H, Sapkota RP. The long-term mental health consequences of torture, loss, and insecurity: A qualitative study among survivors of armed conflict in the Dang district of Nepal. Frontiers in psychiatry. 2020;10. 10.3389/fpsyt.2019.00941 PMC6975134 32009989

[59] Aker J. Cash or Coupons? Testing the Impacts of Cash versus Vouchers in the Democratic Republic of Congo. 2013.

[60] Avdeenko A, Gilligan MJ. Community-Driven Development and Social Capital: Lab-in-the-Field Evidence from Sudan. 2012.

[61] Bamidele S. War, Sex and Justice: Barriers to Gender Justice in Post-Conflict Liberia. Int J Crim Justice Sci. 2017;12(1):69–82.

[62] Barath A. Children’s well-being after the war in Kosovo: survey in 2000. Croatian medical journal. 2002;43(2):199–208. 11885048

[63] Cilliers J, Dube O, Siddiqi B. Can the Wounds of War be Healed?: Experimental Evidence on Reconciliation in Sierra Leone: Center for Global Development; 2015.

[64] Dagnelie O DL G. D. Maystadt J. F. Violence, selection and infant mortality in Congo. Journal of Health Economics. 2018;59:153–77. doi: 10.1016/j.jhealeco.2018.02.004 29753197

[65] Daoud N S K. O’Campo P. Anderson K. Agbaria A. K. Internal displacement and health among the Palestinian minority in Israel. Social science & medicine (1982). 2012;74(8):1163–71. doi: 10.1016/j.socscimed.2011.12.041 22386615

[66] Fearon J, Humphreys M, Weinstein J. Community-driven reconstruction in Lofa County: impact assessment. 2008.

[67] Fearon JD, Humphreys M, Weinstein JM. Can development aid contribute to social cohesion after civil war? Evidence from a field experiment in post-conflict Liberia. American Economic Review. 2009;99(2):287–91.

[68] Fiske L S R. Gender, poverty and violence: Transitional justice responses to converging processes of domination of women in eastern DRC, northern Uganda and Kenya. Women Stud Int Forum. 2015;51:110–7.

[69] Gizelis TI J J. Decoupling local ownership? The lost opportunities for grassroots women’s involvement in Liberian peacebuilding. Cooperation and Conflict. 2016;51(4):539–56.

[70] Glass N R P. Tosha M. Mpanano M. Cinyabuguma M. A Congolese-US participatory action research partnership to rebuild the lives of rape survivors and their families in eastern Democratic Republic of Congo. Global Public Health. 2012;7(2):184–95. doi: 10.1080/17441692.2011.594449 21732709

[71] Humphreys M, De La Sierra RS, Van der Windt P. Social and economic impacts of Tuungane: Final report on the effects of a community driven reconstruction program in Eastern Democratic Republic of Congo. New York, NY: Columbia University; 2012.

[72] Kett ME. Internally displaced peoples in Bosnia-Herzegovina: impacts of long-term displacement on health and well-being. Medicine, conflict, and survival. 2005;21(3):199–215. doi: 10.1080/13623690500166028 16180733

[73] Kim Y DJ Y. H. Caso V. Bushnell C. D. Saposnik G. Countries with women inequalities have higher stroke mortality. International journal of stroke: official journal of the International Stroke Society. 2017;12(8):869–74. doi: 10.1177/1747493017694389 28946830

[74] King E. Can Development Interventions Help Post-conflict Communities Build Social Cohesion? The Case of the Liberia Millennium Villages. 2013.

[75] Kirkby K G G. N. L. Kurinczuk J. J. Rajapakse S. Fernando S. D. Knowledge, attitudes and practices relevant to malaria elimination amongst resettled populations in a post-conflict district of northern Sri Lanka. Trans Roy Soc Trop Med Hyg. 2013;107(2):110–8. doi: 10.1093/trstmh/trs015 23222949

[76] Kyamusugulwa P MH D. Jacobs C. Accountability mechanisms in community-driven reconstruction in eastern Democratic Republic of Congo. Development in practice. 2018;28(1):4–15.

[77] Luitel NPJ, Sapkota M. J. D., Tol R. P., Kohrt W. A., Thapa B. A., Komproe S. B., et al. Conflict and mental health: a cross-sectional epidemiological study in Nepal. Social Psychiatry and Psychiatric Epidemiology. 2013;48(2):183–93. doi: 10.1007/s00127-012-0539-0 22777395

[78] Maclure R D M. Reconstruction versus transformation: Post-war education and the struggle for gender equity in Sierra Leone. International Journal of Educational Development. 2009;29(6):612–20.

[79] Mugisha J M H. Wandiembe P. Kinyanda E. Prevalence and factors associated with Posttraumatic Stress Disorder seven years after the conflict in three districts in northern Uganda (The Wayo-Nero Study). Bmc Psychiatry. 2015;15. 10.1186/s12888-015-0551-5 PMC4513792 26205192

[80] Najafizada S AL R. Bourgeault I. L. Community health workers of Afghanistan: a qualitative study of a national program. Confl Health. 2014;8:26. doi: 10.1186/1752-1505-8-26 25904976 PMC4405840

[81] Palmer J JK A. H. Surur E. I. Checchi F. Jones C. Changing landscapes, changing practice: negotiating access to sleeping sickness services in a post-conflict society. Social science & medicine (1982). 2014;120:396–404. 24679924 10.1016/j.socscimed.2014.03.012

[82] Pavlish C H A. Displaced persons’ perceptions of human rights in Southern Sudan. International Nursing Review. 2009;56(4):416–25. doi: 10.1111/j.1466-7657.2009.00739.x 19930069

[83] Pereira S MB I. du Toit R. Ramke J. Improving access to eye health services in rural Timor-Leste. Rural and Remote Health. 2012;12(3). 22994876

[84] Rivillas J CR R. D. Song G. Martel A. How do we reach the girls and women who are the hardest to reach? Inequitable opportunities in reproductive and maternal health care services in armed conflict and forced displacement settings in Colombia. Plos One. 2018;13(1). doi: 10.1371/journal.pone.0188654 29346375 PMC5773007

[85] Schlecht J R E. Babirye J. Early relationships and marriage in conflict and post-conflict settings: vulnerability of youth in Uganda. Reproductive health matters. 2013;21(41):234–42. doi: 10.1016/S0968-8080(13)41710-X 23684206

[86] Schramm S K F. O. Smedemark S. A. Ovuga E. Sodemann M. Gender and age disparities in adult undernutrition in northern Uganda: high-risk groups not targeted by food aid programmes. Tropical Medicine & International Health. 2016;21(6):807–17. 27102720 10.1111/tmi.12708

[87] Somasundaram D S S. Rebuilding community resilience in a post-war context: developing insight and recommendations—a qualitative study in Northern Sri Lanka. International Journal of Mental Health Systems. 2013;7. 10.1186/1752-4458-7-3 PMC3630062 23305538

[88] Suarez EB. Surviving juntas (together): lessons of resilience of indigenous Quechua women in the aftermath of conflict in Peru. Intervention. 2015;13(1):6–18.

[89] Svoronos T, Macauley RJ, Kruk ME. Can the health system deliver? Determinants of rural Liberians’ confidence in health care. Health Policy and Planning. 2015;30(7):823–9. doi: 10.1093/heapol/czu065 25070742

[90] Tuncalp O F I. S. Phillips S. J. Williams I. Sacko M. Toure O. B. Thomas, et al. Conflict, displacement and sexual and reproductive health services in Mali: analysis of 2013 health resources availability mapping system (HeRAMS) survey. Conflict and Health. 2015;9. doi: 10.1186/s13031-015-0051-8 26379767 PMC4568579

[91] Chiumento A, Rutayisire T, Sarabwe E, Hasan MT, Kasujja R, Nabirinde R, et al. Exploring the mental health and psychosocial problems of Congolese refugees living in refugee settings in Rwanda and Uganda: a rapid qualitative study. Conflict and Health. 2020;14(1). doi: 10.1186/s13031-020-00323-8 33292363 PMC7670672

[92] Costenbader E, Zissette S, Martinez A, LeMasters K, Dagadu NA, Deepan P, et al. Getting to intent: Are social norms influencing intentions to use modern contraception in the DRC? PLoS One. 2019;14(7):e0219617. doi: 10.1371/journal.pone.0219617 31310641 PMC6634398

[93] Meyer C, Evans D, Soucat A, Dkhimi F, Akweongo P, Kessy F, et al. Leaving no one behind? Reaching the informal sector, poor people and marginalised groups with Social Health Protection. Journal of Poverty and Social Justice. 2018;26(3):459–65.

[94] Raven J, Wurie H, Idriss A, Bah AJ, Baba A, Nallo G, et al. How should community health workers in fragile contexts be supported: qualitative evidence from Sierra Leone, Liberia and Democratic Republic of Congo. Human Resources for Health. 2020;18(1). doi: 10.1186/s12960-020-00494-8 32770998 PMC7414260

[95] Roseboom TJ. Why achieving gender equality is of fundamental importance to improve the health and well-being of future generations: a DOHaD perspective. J Dev Orig Health Dis. 2020;11(2):101–4. doi: 10.1017/S2040174419000734 31735185

[96] Buvinic M DG M. Shemyakina O. N. Armed Conflict, Gender, and Schooling. World Bank Econ Rev. 2014;28(2):311–9.

[97] Dunleavy K. Physical therapy education and provision in Cambodia: A framework for choice of systems for development projects. Disabil Rehabil. 2007;29(11–12):903–20. doi: 10.1080/09638280701240433 17577725

[98] Elbadawi IA. Postconflict transitions: An overview. World Bank Econ Rev. 2008;22(1):1–7.

[99] Hynes HP. On the battlefield of women’s bodies: An overview of the harm of war to women. Women Stud Int Forum. 2004;27(5–6):431–45.

[100] Kelly JD, Barrie MB, Ross RA, Temple BA, Moses LM, Bausch DG. Housing equity for health equity: a rights-based approach to the control of Lassa fever in post-war Sierra Leone. Bmc International Health and Human Rights. 2013;13:2. doi: 10.1186/1472-698X-13-2 23282054 PMC3562201

[101] Luta X D T. Kosovo-Serbs’ experiences of seeking healthcare in a post-conflict and ethnically segregated health system. International Journal of Public Health. 2013;58(3):377–83. doi: 10.1007/s00038-012-0403-8 22945841

[102] Maconachie R H G. Re-Thinking the Child Labor "Problem" in Rural sub-Saharan Africa: The Case of Sierra Leone’s Half Shovels. World Development. 2016;78:136–47.

[103] Spalj S, Plancak D, Bozic D, Kasaj A, Willershausen B, Jelusic D. Periodontal conditions and oral hygiene in rural population of post-war Vukovar region, Croatia in correlation to stress. European journal of medical research. 2008;13(3):100–6. 18499555

[104] Toebes B, Stronks K. Closing the gap: a human rights approach towards social determinants of health. european Journal of health law. 2016;23(5):510–24. doi: 10.1163/15718093-12341402 29210249

[105] Ahmed S, Creanga AA, Gillespie DG, Tsui AO. Economic status, education and empowerment: implications for maternal health service utilization in developing countries. PloS one. 2010;5(6):e11190. doi: 10.1371/journal.pone.0011190 20585646 PMC2890410

[106] National Academies of Sciences, Engineering, and Medicine, Health and Medicine Division; Board on Health Care Services, Committee on Health Care Utilization and Adults with Disabilities. Health-Care Utilization as a Proxy in Disability Determination. 2018. In: National Academies of Sciences, Engineering, and Medicine [Internet]. Washington, DC: The National Academies Press; [160]. https://www.nap.edu/catalog/24969/health-care-utilization-as-a-proxy-in-disability-determination. 29782136

[107] Recommendation 1074 on family policy, 3 May 1988. Annual review of population law. 1988;15:83, 471–4. 12289698

[108] Loevinsohn B, Harding A. Buying results? Contracting for health service delivery in developing countries. The Lancet. 2005;366(9486):676–81. doi: 10.1016/S0140-6736(05)67140-1 16112305

[109] Newbrander W. Rebuilding health systems and providing health services in fragile states. Occasional Papers. 2007;7. 10.1111/j.1467-7717.2010.01235.x 21913929

[110] Pfeiffer J, Johnson W, Fort M, Shakow A, Hagopian A, Gloyd S, et al. Strengthening health systems in poor countries: a code of conduct for nongovernmental organizations. American journal of public health. 2008;98(12):2134–40. doi: 10.2105/AJPH.2007.125989 18923125 PMC2636539

[111] Copelon R. Gendered war crimes: Reconceptualizing rape in time of war. Women’s rights, human rights: International feminist perspectives. 1995:197–214.

[112] Whitbread J. Mainstreaming gender in conflict reduction: from challenge to opportunity. Gender & Development. 2004;12(3):41–9.

[113] Co-operation OfE, Development. Conflict and Fragility: Armed Violence Reduction: Enabling Development: OECD Pub.; 2009.

[114] Palmer CA, Zwi AB. Women, health and humanitarian aid in conflict. Disasters. 1998;22(3):236–49. doi: 10.1111/1467-7717.00089 9753813

[115] Broaddus-Shea ET, Kobeissi L, Ummer O, Say L. A systematic review of monitoring and evaluation indicators for sexual and reproductive health in humanitarian settings. Conflict and Health. 2019;13(1):43. doi: 10.1186/s13031-019-0221-1 31636697 PMC6791013

[116] Sorenson B. Women and Post-conflict Reconstruction: Issues and sources. War-Torn Societies Project Occasional Paper 3. 1998.

[117] Maher D, Waswa L, Baisley K, Karabarinde A, Unwin N, Grosskurth H. Distribution of hyperglycaemia and related cardiovascular disease risk factors in low-income countries: a cross-sectional population-based survey in rural Uganda. International Journal of Epidemiology. 2010;40(1):160–71. doi: 10.1093/ije/dyq156 20926371 PMC3043279

[118] UBOS, ICF. International Inc: Uganda Demographic and Health Survey 2011. Kampala, Uganda. 2012.

[119] Centre HS. Human security report 2005: war and peace in the 21st century: Oxford University Press; 2005.

[120] Doherty S, Hulland E, Lopes-Cardozo B, Kirupakaran S, Surenthirakumaran R, Cookson S, et al. Prevalence of mental disorders and epidemiological associations in post-conflict primary care attendees: A cross-sectional study in the Northern Province of Sri Lanka. BMC Psychiatry. 2019;19. doi: 10.1186/s12888-019-2064-0 30832646 PMC6399832

[121] Roberts B, Odong VN, Browne J, Ocaka KF, Geissler W, Sondorp E. An exploration of social determinants of health amongst internally displaced persons in northern Uganda. Conflict and Health. 2009;3(1):10. doi: 10.1186/1752-1505-3-10 20003516 PMC2803159

[122] Elzein HL, Ammar DF. Parent and teacher perceptions of assessing Lebanese children’s reaction to war-related stress: A survey of psychological and behavioral functioning. Journal of Child & Adolescent Trauma. 2010;3(4):255–78.

[123] Joshi PT, O’donnell DA. Consequences of child exposure to war and terrorism. Clinical child and family psychology review. 2003;6(4):275–92. doi: 10.1023/b:ccfp.0000006294.88201.68 14719639

[124] Guttmann-Steinmetz S, Shoshani A, Farhan K, Aliman M, Hirschberger G. Living in the crossfire: Effects of exposure to political violence on Palestinian and Israeli mothers and children. International Journal of Behavioral Development. 2012;36(1):71–8.

[125] Finzi-Dottan R, Dekel R, Lavi T, Su’ali T. Posttraumatic stress disorder reactions among children with learning disabilities exposed to terror attacks. Comprehensive psychiatry. 2006;47(2):144–51. doi: 10.1016/j.comppsych.2005.05.001 16490573

[126] Pat-Horenczyk R, Qasrawi R, Lesack R, Haj-Yahia M, Peled O, Shaheen M, et al. Posttraumatic Symptoms, Functional Impairment, and Coping among Adolescents on Both Sides of the Israeli–Palestinian Conflict: A Cross-Cultural Approach. Applied Psychology. 2009;58(4):688–708.

[127] Dyregrov A, Gupta L, Gjestad R, Mukanoheli E. Trauma exposure and psychological reactions to genocide among Rwandan children. Journal of traumatic stress. 2000;13(1):3–21. doi: 10.1023/A:1007759112499 10761171

[128] Gupta L, Zimmer C. Psychosocial intervention for war-affected children in Sierra Leone. The British Journal of Psychiatry. 2008;192(3):212–6. doi: 10.1192/bjp.bp.107.038182 18310582

[129] Lonigan CJ, Phillips BM, Richey JA. Posttraumatic stress disorder in children: diagnosis, assessment, and associated features. Child and Adolescent Psychiatric Clinics of North America. 2003. 10.1016/s1056-4993(02)00105-0 12725007

[130] Slone M, Shechner T. Psychiatric consequences for Israeli adolescents of protracted political violence: 1998–2004. Journal of Child Psychology and Psychiatry. 2009;50(3):280–9. doi: 10.1111/j.1469-7610.2008.01940.x 18665881

[131] Jagodić GK, Kontak K. Normalization: A key to children’s recovery. Children and disasters: A practical guide to healing and recovery: Oxford University Press; 2002.

[132] Hyder AA, Maman S, Nyoni JE, Khasiani SA, Teoh N, Premji Z, et al. The pervasive triad of food security, gender inequity and women’s health: exploratory research from sub-Saharan Africa. Afr Health Sci. 2005;5(4):328–34. doi: 10.5555/afhs.2005.5.4.328 16615845 PMC1831953

[133] Maru DS-R, Andrews J, Schwarz D, Schwarz R, Acharya B, Ramaiya A, et al. Crossing the quality chasm in resource-limited settings. Globalization and health. 2012;8(1):41. doi: 10.1186/1744-8603-8-41 23193968 PMC3526495

[134] Tedrow VA, Zelaya CE, Kennedy CE, Morin SF, Khumalo-Sakutukwa G, Sweat MD, et al. No “magic bullet”: exploring community mobilization strategies used in a multi-site community based randomized controlled trial: Project Accept (HPTN 043). AIDS and behavior. 2012;16(5):1217–26. doi: 10.1007/s10461-011-0009-9 21822627 PMC3400971

[135] Garwood P. Pakistan, Afghanistan look to women to improve health care. Bulletin of the World Health Organization. 2006;84:845–7. 17143454 PMC2627542

[136] Harris M, Haines A. Brazil’s family health programme. BMJ (Clinical research ed). 2010;341(c4945):1171–76. doi: 10.1136/bmj.c4945 21115587

[137] Javanparast S, Baum F, Labonte R, Sanders D. Community health workers’ perspectives on their contribution to rural health and well-being in Iran. American journal of public health. 2011;101(12):2287–92. doi: 10.2105/AJPH.2011.300355 22021303 PMC3222427

[138] Sebhatu A. The implementation of Ethiopia’s Health Extension Program: an overview. Addis Ababa, Ethiopia. 2008.

[139] Ameli O, Newbrander W. Contracting for health services: effects of utilization and quality on the costs of the Basic Package of Health Services in Afghanistan. Bulletin of the World Health Organization. 2008;86:920–8. doi: 10.2471/blt.08.053108 19142292 PMC2649585

[140] Peters DH, Noor AA, Singh LP, Kakar FK, Hansen PM, Burnham G. A balanced scorecard for health services in Afghanistan. Bulletin of the World Health Organization. 2007;85:146–51. doi: 10.2471/blt.06.033746 17308736 PMC2636277

