# Supplementary material for: Health inequalities in post-conflict settings: A systematic review
Source: PLoS One. 2022 Mar 14;17(3):e0265038. doi: 10.1371/journal.pone.0265038 (PMC8920275; doi:10.1371/journal.pone.0265038)
Supplement: S1 Fig — (DOC) [file pone.0265038.s002.doc]

**S1 Fig. PRISMA Flow Diagram of Selection Process**

The flow chart below illustrates the methods used for systematic literature search.

Studies deemed appropriate for screening **(n=1,226)**

**Screening**

**Included**

**Eligibility**

**Identification**

Number of full-text articles retrieved and assessed for eligibility **(n = 340)**

Number of records identiﬁed through searching electronic databases **(n=4,742)** search strategy: PubMed (**n =3,285**);

WoS (**n = 803**) & PsycInfo (**n = 654**).

Number of additional records identified through other sources, mainly 3 i.e. database (**n =107**).

Only **7** studies were included based on in/excl. criteria

Full-text articles excluded (**n= 285**), with reasons:

Not focused on Post-war, health, inequality concepts or PROGRESS PLUS factors.

Unclear study design and or methodology, no focus on post-conflict settings), articles about

mass killings and/or natural disasters.

Number of records after duplicates removed

(**n = 2,999**)

Studies excluded **(n = 1,773)** after titles screening because their titles did not include post-war, health, and/or inequality.

Studies excluded **(n= 886)** on the basis of abstracts (abstracts did not focus on inequality in relation to post war and health)

Duplicate records removed **(n= 1,743)**

Potentially appropriate studies to be included in the analysis **(n = 55)**

Studies included in the analysis:

**(n= 55)** from above databases & **(n= 7)** from 3i.e. database

**(n = 62)**
