# Supplementary material for: Health inequalities in post-conflict settings: A systematic review
Source: PLoS One. 2022 Mar 14;17(3):e0265038. doi: 10.1371/journal.pone.0265038 (PMC8920275; doi:10.1371/journal.pone.0265038)
Supplement: S1 File — (DOCX) [file pone.0265038.s007.docx]

**S1 File. Eligibility criteria**

| **Inclusion criterion** | **Exclusion criterion** |
| --- | --- |
| Contain at least one of the three  key concepts:   - Post-war - Inequality - Health | The focus of the study is not at least one of the three key concepts:   - Not about post-war - Not about inequality - Not about health |
| Contain at least one of the eight PROGRESS-Plus acronyms of interest:   - Place of residence; - Race, ethnicity, culture, language; - Occupation; - Gender and sex; - Religion; - Education; - Socio-economic Status - Social Capital | Focus is not about at least one of the eight PROGRESS-Plus acronyms of interest |
| Studies with a focus on:   - Study setting = post-conflict environment; - Study participants = the most vulnerable population sub-groups living in post-conflict areas such as the civilian population, women, children, internally displaced persons (IDPs), and people with symptoms of mental illness. - Study interventions = assessing/addressing the structural determinants of health inequalities - Study methodology = qualitative, quantitative, or mixed methods | Conference or Poster abstracts and studies on serving or former military personnel.  Studies about ongoing conflict settings  Articles about mass killings, genocide, and natural disasters.  Unclear study design and or methodology |
| Peer-reviewed articles  No language restriction | Abstract-only studies  Conference abstracts  Studies on serving or former military personnel  Reviews-only studies |
