# Supplementary material for: Health inequalities in post-conflict settings: A systematic review
Source: PLoS One. 2022 Mar 14;17(3):e0265038. doi: 10.1371/journal.pone.0265038 (PMC8920275; doi:10.1371/journal.pone.0265038)
Supplement: S2 File — (DOCX) [file pone.0265038.s008.docx]

**S2 File. Key concepts and search terms**

| **Post-war** | **Inequality** | **Health** | **PROGRESS-PLUS Factors** |
| --- | --- | --- | --- |
| "Post war" | Inequality | Health | Place of residence |
| "After war" | Inequalities | Healthcare | Race, ethnicity, culture, language |
| "Post-conflict" | Equality | "Health access" | Occupation |
| "After conflict" | Equity | "Access to health" | Gender and sex |
| "Post violence" |  | "Healthcare delivery" | Religion |
| "After violence" |  | "Healthcare system" | Education |
|  |  | "Healthcare distribution" | Socio-economic Status |
|  |  | Well-being | Social Capital |
|  |  | Wellbeing |  |
|  |  | Mortality |  |
|  |  | Morbidity |  |
|  |  | "Food intake" |  |
|  |  | Reconstruction |  |
|  |  | "Progressive realization" |  |
|  |  | Infrastructure |  |
|  |  | Distribution |  |
|  |  | Barriers |  |
|  |  | Enablers |  |
|  |  | Facilitators |  |
|  |  | Facility |  |
|  |  | Facilities |  |
