# Supplementary material for: Health inequalities in post-conflict settings: A systematic review
Source: PLoS One. 2022 Mar 14;17(3):e0265038. doi: 10.1371/journal.pone.0265038 (PMC8920275; doi:10.1371/journal.pone.0265038)
Supplement: S3 File — (DOCX) [file pone.0265038.s009.docx]

**S3 File. Web of Science search strategy**

| **Concept 1- Post War**#1 | TOPIC: (("Post War") OR ("Post Conflict") OR ("Post violence") OR ("After war") OR ("After conflict") OR ("After violence")) | **14,106**  *Timespan 2018-2020 = 3,625* |
| --- | --- | --- |
| **Concept 2: Inequality**#2 | TOPIC: (("Inequality") OR ("Inequalities") OR ("Equality") OR ("Equity")) | **346,266**  *Timespan 2018-2020 =* *88,154* |
| **Concept 3: Health**# 3 | TOPIC: (("Health") OR ("Healthcare") OR ("health access") OR ("Access to health") OR ("Healthcare delivery") OR ("Healthcare system") OR ("Healthcare distribution") OR ("Well-being") OR ("Wellbeing") OR ("Mortality") OR ("Morbidity”) OR ("Food intake") 0R (Reconstruction) OR ("Progressive realization") OR (“Infrastructure”) OR (“Distribution”) OR (“Barriers”) OR (“Enablers”) OR (“Facilitators”) OR (“Facility”) OR ("Facilities")) | **6,163,176**  *Timespan 2018-2020 =* *1,402,481* |
| **Concept 4: PROGRESS Plus factors**# 4 | TOPIC: ((("Place of residence") OR ("Race, ethnicity, culture, language") OR ("Occupation") OR ("Gender and sex") OR ("Religion") OR ("Education") OR ("Socio-economic Status") OR ("Social Capital"))) | **1,116,776**  *Timespan 2018-2020 =* *289,455* |
| Combinations of Concept 1- Post War AND Concept 2-Inequality AND Concept 3- Health AND Concept 4: PROGRESS PLUS Factors: #1 AND #2 AND #3 AND #4 | You searched for: #4 AND #3 AND #2 AND #1 **(7,185,207 articles)**  Timespan: All years. Indexes: SCI-EXPANDED, SSCI, A&HCI, CPCI-S, CPCI-SSH, ESCI.  ("Post War") OR ("Post Conflict") OR ("Post violence") OR ("After war") OR ("After conflict") OR ("After violence") AND ("Inequality") OR ("Inequalities") OR ("Equality") OR ("Equity") AND (("Health") OR ("Healthcare") OR ("health access") OR ("Access to health") OR ("Healthcare delivery") OR ("Healthcare system") OR ("Healthcare distribution") OR ("Well-being") OR ("Wellbeing") OR ("Mortality") OR ("Morbidity”) OR ("Food intake") 0R (Reconstruction) OR ("Progressive realization") OR (“Infrastructure”) OR (“Distribution”) OR (“Barriers”) OR (“Enablers”) OR (“Facilitators”) OR (“Facility”) OR ("Facilities")) AND ("Place of residence") OR ("Race, ethnicity, culture, language”) OR ("Occupation") OR ("Gender and sex") OR ("Religion") OR ("Education") OR ("Socio-economic Status") OR ("Social Capital"). | **32**  *Timespan 2018-2020=* *12* |

Search: 32 results (from Web of Science Core Collection)
