# Supplementary material for: Health inequalities in post-conflict settings: A systematic review
Source: PLoS One. 2022 Mar 14;17(3):e0265038. doi: 10.1371/journal.pone.0265038 (PMC8920275; doi:10.1371/journal.pone.0265038)
Supplement: Attachment — Submitted filename: Response to Reviewers.docx [file pone.0265038.s010.docx]

**Rebuttal letter**

**Response to Reviewers**

PONE-D-21-08382

**Health inequalities in post-conflict settings: A systematic review.**

PLOS ONE

Dear Editor,

We would like to thank you and the reviewers for evaluating our manuscript and providing constructive comments. We have carefully considered the points raised during the review process and have tried to address every one of them. We have copied the review decision from the submission menu of the editorial manager and used this format to write our responses. Below we provide the point-by-point responses to the comments. All modifications in the revised manuscript have been highlighted (with Track Changes). Of course, we remain at your disposal should you have any further suggestions for improvements of our paper.

Dear Dr. Bwirire,

Thank you for submitting your manuscript to PLOS ONE. After careful consideration, we feel that it has merit but does not fully meet PLOS ONE’s publication criteria as it currently stands. Therefore, we invite you to submit a revised version of the manuscript that addresses the points raised during the review process.

Please ensure that your decision is justified on PLOS ONE’s [publication criteria](https://journals.plos.org/plosone/s/criteria-for-publication) and not, for example, on novelty or perceived impact.

Please submit your revised manuscript by Oct 03 2021 11:59 PM. If you will need more time than this to complete your revisions, please reply to this message or contact the journal office at [plosone@plos.org](mailto:plosone@plos.org). When you're ready to submit your revision, log on to <https://www.editorialmanager.com/pone/> and select the 'Submissions Needing Revision' folder to locate your manuscript file.

Please include the following items when submitting your revised manuscript:

- A rebuttal letter that responds to each point raised by the academic editor and reviewer(s). You should upload this letter as a separate file labeled 'Response to Reviewers'.
- A marked-up copy of your manuscript that highlights changes made to the original version. You should upload this as a separate file labeled 'Revised Manuscript with Track Changes'.
- An unmarked version of your revised paper without tracked changes. You should upload this as a separate file labeled 'Manuscript'.

If you would like to make changes to your financial disclosure, please include your updated statement in your cover letter. Guidelines for resubmitting your figure files are available below the reviewer comments at the end of this letter.

If applicable, we recommend that you deposit your laboratory protocols in protocols.io to enhance the reproducibility of your results. Protocols.io assigns your protocol its own identifier (DOI) so that it can be cited independently in the future. For instructions see: <http://journals.plos.org/plosone/s/submission-guidelines#loc-laboratory-protocols>. Additionally, PLOS ONE offers an option for publishing peer-reviewed Lab Protocol articles, which describe protocols hosted on protocols.io. Read more information on sharing protocols at <https://plos.org/protocols?utm_medium=editorial-email&utm_source=authorletters&utm_campaign=protocols>.

We look forward to receiving your revised manuscript.

Kind regards,

Jakob Pietschnig, PhD, MSc
Academic Editor
PLOS ONE

Journal Requirements:

When submitting your revision, we need you to address these additional requirements.

1. Please ensure that your manuscript meets PLOS ONE's style requirements, including those for file naming. The PLOS ONE style templates can be found at 
<https://journals.plos.org/plosone/s/file?id=wjVg/PLOSOne_formatting_sample_main_body.pdf> and 
<https://journals.plos.org/plosone/s/file?id=ba62/PLOSOne_formatting_sample_title_authors_affiliations.pdf>

>>> **Author Response:** Thank you very much for your constructive feedback! Accordingly, we went through the entire manuscript to ensure it meets PLOS ONE’s style requirements. Additionally, the file naming has been revised in line with the style template.

2. Please review your reference list to ensure that it is complete and correct. If you have cited papers that have been retracted, please include the rationale for doing so in the manuscript text, or remove these references and replace them with relevant current references. Any changes to the reference list should be mentioned in the rebuttal letter that accompanies your revised manuscript. If you need to cite a retracted article, indicate the article’s retracted status in the References list and also include a citation and full reference for the retraction notice.

>>> **Author Response:** Thank you for pointing this out. We have done so accordingly.

3. Please confirm that you have included all items recommended in the PRISMA checklist including:

- a Supplemental file of the results of the individual components of the quality assessment, not just the overall score, for each study included.

**>>> Author response:** As suggested by the reviewer, we have created a Supplemental file with the results of the individual components of the quality assessment for each study included. It is named S4 Table. Individual Quality Assessment for each study included

- details of reasons for study exclusions in the PRISMA flowchart and number of studies excluded for each reason.

**>>> Author response:** As suggested by the reviewer, we have provided the details of the reasons for study exclusions in the PRISMA flowchart and the number of studies excluded for each reason.

- See <https://journals.plos.org/plosmedicine/article?id=10.1371/journal.pmed.1000100#pmed-1000100-t003> for guidance on reporting.

Thank you.

**>>> Author response:** We have done so accordingly, following additional journal requirements kindly shared by the reviewer in the following article: “The PRISMA Statement for Reporting Systematic Reviews and Meta-Analyses of Studies that Evaluate Health Care Interventions: Explanation and Elaboration”

4. We note that you have stated that you will provide repository information for your data at acceptance. Should your manuscript be accepted for publication, we will hold it until you provide the relevant accession numbers or DOIs necessary to access your data. If you wish to make changes to your Data Availability statement, please describe these changes in your cover letter and we will update your Data Availability statement to reflect the information you provide.

**>>> Author response:** Thank you for pointing this out. All extracted data that was used in this review are available in the predesigned data extraction sheet which is referred to in the supplementary material as S1 Table. Data extraction sheet information from selected studies.

5. Please remove all personal information, ensure that the data shared are in accordance with participant consent, and re-upload a fully anonymized data set.

Note: spreadsheet columns with personal information must be removed and not hidden as all hidden columns will appear in the published file.

Additional guidance on preparing raw data for publication can be found in our Data Policy (<https://journals.plos.org/plosone/s/data-availability#loc-human-research-participant-data-and-other-sensitive-data>) and in the following article: <http://www.bmj.com/content/340/bmj.c181.long>.

**>>> Author response:** Thank you for pointing this out. The data in Supplemental File (S1 Table) does not contain any personal information.

6. Thank you for submitting the above manuscript to PLOS ONE. During our internal evaluation of the manuscript, we found significant text overlap between your submission and the following previously published works, some of which you are an author.

<https://www.scribd.com/document/328300724/Estadisticas-de-Salud-Mundial>
<https://applications.emro.who.int/dsaf/dsa955.pdf>
<https://journals.plos.org/plosone/article?id=10.1371%2Fjournal.pone.0210071>
<https://www.who.int/social_determinants/links/events/conflicts_and_sdh_emro_revison_06_2007.pdf>
<https://apps.who.int/disasters/repo/8678.pdf>
<https://www.pure.ed.ac.uk/ws/files/19589731/Palmer_J._2014_._Changing_landscapes_changing_practice.pdf>
<https://www.coe.int/en/web/genderequality/what-is-gender-mainstreaming>
<https://onlinelibrary.wiley.com/doi/pdf/10.1111/tmi.12708>
<https://www.raiseinitiative.org/library/download.php?id=495>
<https://www.tandfonline.com/doi/abs/10.1080/17441692.2013.819587?journalCode=rgph20>

We would like to make you aware that copying extracts from previous publications, especially outside the methods section, word-for-word is unacceptable. In addition, the reproduction of text from published reports has implications for the copyright that may apply to the publications.

Please revise the manuscript to rephrase the duplicated text, cite your sources, and provide details as to how the current manuscript advances on previous work. Please note that further consideration is dependent on the submission of a manuscript that addresses these concerns about the overlap in text with published work.

We will carefully review your manuscript upon resubmission, so please ensure that your revision is thorough.

[Note: HTML markup is below. Please do not edit.]

**>>> Author response:** Thank you for your comments and suggestions. We have done so accordingly.

**Reviewers' comments:

Reviewer's Responses to Questions**

**Comments to the Author**

1. Is the manuscript technically sound, and do the data support the conclusions?

The manuscript must describe a technically sound piece of scientific research with data that supports the conclusions. Experiments must have been conducted rigorously, with appropriate controls, replication, and sample sizes. The conclusions must be drawn appropriately based on the data presented.

Reviewer #1: Yes

Reviewer #2: Yes

Reviewer #3: Yes

2. Has the statistical analysis been performed appropriately and rigorously?

Reviewer #1: Yes

Reviewer #2: N/A

Reviewer #3: Yes

3. Have the authors made all data underlying the findings in their manuscript fully available?

The [PLOS Data policy](http://www.plosone.org/static/policies.action#sharing) requires authors to make all data underlying the findings described in their manuscript fully available without restriction, with rare exception (please refer to the Data Availability Statement in the manuscript PDF file). The data should be provided as part of the manuscript or its supporting information, or deposited to a public repository. For example, in addition to summary statistics, the data points behind means, medians and variance measures should be available. If there are restrictions on publicly sharing data—e.g. participant privacy or use of data from a third party—those must be specified.

Reviewer #1: Yes

Reviewer #2: Yes

Reviewer #3: Yes

 4. Is the manuscript presented in an intelligible fashion and written in standard English?

PLOS ONE does not copyedit accepted manuscripts, so the language in submitted articles must be clear, correct, and unambiguous. Any typographical or grammatical errors should be corrected at revision, so please note any specific errors here.

Reviewer #1: Yes

Reviewer #2: Yes

Reviewer #3: Yes

5. Review Comments to the Author

Please use the space provided to explain your answers to the questions above. You may also include additional comments for the author, including concerns about dual publication, research ethics, or publication ethics. (Please upload your review as an attachment if it exceeds 20,000 characters)

**Reviewer #1: Dear Editors,**

Thank you for the opportunity to review this systematic review of research on health inequalities in post-conflict settings.

The article has many merits as it stands now. Relevant concepts (such as health inequalities, post-conflict, PROGRESS-Plus) are clearly defined. The aims of the study are appropriate, relevant, and narrow enough for the systematic review. My impression is that the authors conducted a rigorous, unbiased and comprehensive search for all studies according to the search strategy, which was clearly described and seems suitable for the aims.

Given the good shape of the article in its current condition, I believe the authors are capable of submitting an improved revised manuscript.

**Response to Reviewer #1**

Thank you very much for agreeing with us on the aim of our manuscript and recognizing that it has many merits as it stands now. Furthermore, we have read your comments carefully and tried our best to address them one by one by providing a detailed explanation of our way of working. By implementing your suggestions, as described below, we hope that you share our view that the manuscript has been improved after this revision.

Some minor but important improvements are suggested below.

1) On page 6, the authors write “… the population of interest consisted of the most vulnerable population groups [34, 35] living in post-conflict areas such as women, children, internally displaced persons (IDPs), and the mentally ill – as these are generally affected the most.” Why were these groups identified as most vulnerable? In the results section it appears as if this choice was data-driven (e.g. “It is widely recognized that armed conflict has a particularly negative impact on women's lives [105, 106]” on page 13 and the paragraph starting with “Women and men have different needs, living conditions, and circumstances, including unequal access to and control over power, resources, human rights, and institutions, including the justice system” on page 21). But the earlier statement on page 6 makes it sound like it is theory-driven. Some clarification as to what guided the sub-group selection would be helpful.

**>>> Author response:** Thank you very much for the great question. Indeed, it might be difficult for the reader to understand what guided the sub-group selection during this review. Putting the sub-group selection process within the broad context of our review, we aimed to collate the current knowledge and knowledge gaps about structural determinants of health inequalities in post-conflict settings. Our understanding is that it is important to identify the characteristics of post-conflict environments that create vulnerability because they either create or contribute towards losses, which lead to vulnerability and negative well-being. Indeed, beforehand the majority of the most vulnerable population sub-groups were already identified. These included Women, Children, IDPs, and People with symptoms of mental symptoms. While applying a stepwise data extraction approach, we found an additional sub-group of interest which consisted of the general population and added this afterward to the final sub-group selection. To help the reader, we have slightly changed the text on page 6 as follows:

“… the population of interest consisted of the most vulnerable population groups [34, 35] living in post-conflict areas such as the general population, women, children, internally displaced persons (IDPs), and the people with symptoms of mental illness – as these are generally affected the most.”

I agree children, IPDs and the mentally ill are likely particularly vulnerable, but why are women included? Since women may make up far more than 50 percent of the population in post-conflict settings due to high male battle-related mortality, what is the logic behind identifying them as one of the most vulnerable groups? Feminist security scholars such as Cynthia Enloe have criticized the merged category “womenandchildren” for removing women’s agency by only portraying them as mindless victims. While I do not necessarily disagree that women are a vulnerable group, a clearer explanation to this effect would help.

**>>> Author response:** Thank you very much for agreeing with us that children, IDPs, and people with symptoms of mental illness are particularly vulnerable in this environment and therefore should be part of the sub-group selection. As noted previously, the final sub-group selection was based on a combination of both: a beforehand selection of the most sub-groups (including women) and an afterward selection of the general population sub-group.

While we tend to agree with your comment about the Feminist security scholars who have criticized the merged category “women and children” for removing women’s agency by only portraying them as mindless victims. We would not feel comfortable discussing the most vulnerable sub-groups in a post-conflict environment without giving proper attention to women and we have addressed this in the revised version of the manuscript: “In particular, we would not feel comfortable discussing the most vulnerable sub-groups in this environment without giving proper attention to women. The reasons for this are multifold: 1) traditional networks that form the foundation of women’s social and family roles may be disrupted during the conflict, 2) women may also leave their communities and husbands for other partners to guarantee a source of food for themselves and their families, and 3) women have to assume the role of the primary breadwinners and caretakers of their families – putting them more at risk [37, 38].” For these reasons, we prefer to point the reader in this direction and maintain the women sub-group in the most vulnerable study population group.

A specific focus on women also risks sidelining men from post-conflict interventions, as argued on page 21. The study from Uganda [86] suggests that women were more likely to be widowed, which also likely means that men are more likely to die in war. Is that not also a vulnerability? Additionally, past research has shown that if post-war aid only focuses on women, it may drive intimate partner violence perpetrated by men who have lost their socioeconomic position as main breadwinners. See Sengupta, Anasuya & Muriel Calo (2016) Shifting gender roles: an analysis of violence against women in post-conflict Uganda. Development in Practice 26 (3), 285–297. It connects to the statement on page 21 that “health services should be available to users in proportion to need (rather than social status)”.

**>>> Author response:** Thank you very much for your constructive feedback! Although we can agree in general with the reviewer’s assessment that both women and men are vulnerable in this environment, we still want to explain why women are most vulnerable than men in post-conflict settings. First, women and men have different exposures to situations that affect their health and access to health care, they also have differential power to influence decisions regarding the provision of health services. In particular, Sorenson (1998) documented one feature of post-conflict environments that is often ignored both by academics as well as policymakers which is the situation of women. Second, in environments where the involvement of women in the labor market is not the norm, women either remain unemployed or become underemployed. According to Bodewig (2002), because women may not be exceptionally educated, even the employment opportunities they could avail would be low-paying jobs.

We finally want to clarify that by giving a bit more attention to women's needs in this environment, our intention was not to take men’s needs away from post-conflict interventions. We only want to specify that women have different roles and perspectives from those men have.

The involvement of women and men at all stages in a response to post-conflict situations is desirable and beneficial.

Aren’t HBTQIA+ individuals a vulnerable group, due to the lack of appropriate health care overall and stigma (that may be heightened due to militarized masculinity in post-war settings)? Why were not these included? Or refugees displaced to other countries?

**>>> Author response:** Thank you very much for these great questions. The reviewer is most likely referring to the LGBTQ+ (which stands for Lesbian, Gay, Bisexual, Transgender, and [Queer](https://en.wikipedia.org/wiki/Queer)) community. Here again, we can agree with the reviewer’s assessment that both the LGBTQ+ community as well as refugees are vulnerable populations in post-conflict environments.

As for the LGBTQ+ community, it was not specified beforehand as one of the most vulnerable sub-groups in this environment. Because of the already existing “LGBTQ discrimination”, their identification (based on sexual orientation or gender identity) would not be easy in practice and maybe could reinforce discrimination that is already present. For this reason, we believe it could have been hard to tell something about this sub-group based on currently available data. However, we certainly believe more attention should be paid to structural inequalities that keep the LGBTQ+ community apart, and that has been highlighted in post-conflict settings. For now, we prefer to point the reader in the direction of the subgroups we have identified as being most vulnerable in this environment (as explained above).

Regarding refugees, we have presented information on page 3 of our manuscript as follows: “For example, children and young people often comprise the highest proportion of the population in refugee and internally displaced persons (IDP) camps and are exposed to risks over which they have little control [6, 7].” By doing so, we acknowledge that it is a vulnerable group but not the most vulnerable one. On the other hand, when people cross an international border to find safety during a crisis, they come under the protection of the UNHCR. That means they can access the funding and resources of the UN. Decades of global news coverage have shown that [life for refugees is difficult](https://preemptivelove.org/blog/what-is-a-refugee/). But they do have some protections, often have access to camps built to provide shelter and food. However, we were ultimately interested in the Internally Displaced Persons (IDPs), as this sub-group stays within their own country and remains under the protection of their government, even if that government is the reason for their displacement. When people flee home but don’t cross an international border to find safety, they remain under the protection of their own country. It may seem like a better situation to find a temporary home within your country of origin. But in reality, it’s often a much more difficult situation. A country at war has extremely limited resources to help displaced people. And internally displaced persons are not protected under the UN. IDPs have not crossed the border to find safety and are therefore amongst the most vulnerable in the world because they often move to areas where it is difficult to deliver humanitarian assistance. Often, they are sheltered among the poorest communities, sharing scarce resources with host communities for years. Therefore, it can be an added value to make a distinction in how to respond to the needs of refugees or internally displaced people (IDPs), and provide critical services to these groups in vulnerable situations to ensure they can live with the safety and dignity they deserve.

2) While I appreciate the clear distinctions of inequality vs. inequity, it seems that the authors sometimes use the terms interchangeably despite having stated the difference between the two terms. For example, on page 7 in this sentence: “We used the PROGRESS-Plus as an organizing framework to assess the structural determinants of health inequalities in post-conflict settings. Using PROGRESS-Plus enables the inclusion of a range of factors and circumvents reliance on any single measure to assess inequities, as this is insufficient.” It’s not always clear to me which of the concepts the authors actually want to explore. Relatedly, why were equality AND inequality included as search terms, but only equity and not inequity (according to Table S6)?

**>>> Author response:** Thank you very much for appreciating our efforts to explain both terms: inequality and inequity. Indeed, by the fact that in languages (including English) where the two terms exist, they are often used interchangeably. Generally, the technical distinction between the two concepts of health *in*equality and health inequity is that health inequality can refer to all differences, and health inequity refers just to those differences which are unnecessary, avoidable, unfair, and unjust. To avoid confusion, we have provided the meaning of each concept. Accordingly, we have revised both concepts throughout our manuscript to help the reader better understand which of the two concepts the authors want to explore.

Finally, inequity was not included as a separate key concept and search term because it refers to a specific type of health inequality that denotes an unjust difference in health. As mentioned previously, this aspect is covered by health inequality as this concept refers to all differences?

Per supplemental Table 6. health inequality was included in the key concepts and search terms for our review. To mitigate the risk of whether we might have missed certain evidence, a significant effort was made by applying a rigorous search strategy (including the use of ineq*) and identifying articles from scientific and professional databases only (Page 29 of the revised manuscript).
3) Some clarifications with respect to conflict vs. post-conflict are needed.

I appreciated the predetermined criteria for inclusion and exclusion. But I would add studies about ongoing conflict settings to the exclusion criteria if those were indeed excluded, in the parenthesis “(e.g., natural disasters/post-tsunami, terror victims, intimate partner violence)” on page 8.

**>>> Author response:** Thank you very much for your constructive feedback. We have addressed your comments as described below: “Abstract-only studies, conference abstracts, studies about ongoing conflict settings, and studies on serving or former military personnel were excluded.”

The authors engage appropriately with the existing literature, for example by discussing the lack of consensus on what constitutes post-conflict. But does Colombia really count as a post-conflict setting? One of the studies included in analysis (Alzate, 2008) was published before the peace agreement between the government and FARC was signed in 2016, and since then the levels of violence have risen above pre-treaty levels. Several armed groups (including organized crime networks and the ELN guerrilla) are still actively fighting. What is the logic behind this inclusion?

**>>> Author response:** Thank you very much for this great question and for agreeing with us on the efforts to clarify what constitutes a post-conflict setting. As explained on page 4 of our manuscript, there is no consensus in the literature about the definition of post-conflict settings. Post-conflict describes the **period immediately after a conflict is over**. But, the term is more difficult to define for many reasons. Firstly, conflicts do not necessarily end with the signing of official peace agreements. Secondly, low-intensity conflict replaces violent conflict because one or several actors are either excluded or not content with the peace agreement. The end of a conflict and subsequently the start of the post-conflict phase, therefore, become difficult to determine. For these reasons, we have elaborated on what we understand under post-conflict settings and, we have also described what characterizes this environment. Specifically for Colombia, this country is a good example of a post-conflict situation. Although the peace agreement was not signed yet, the other characteristics of a post-conflict setting were present (as described on Page 5 of the revised manuscript).

The final selection of Alzate’s paper (The sexual and reproductive rights of internally displaced women: the embodiment of Colombia's crisis) was based on the fact that this paper met all criteria to be included in the final review. The fact that all of the post-conflict characteristics were not present simultaneously was not an issue as – at the time of the final paper selection, Colombia was at a stage of recovery from conflict or crisis and a stage of rebuilding and reconstruction starting from emergency and stabilization followed by transition and recovery, and peace and development.

Are there other studies included on active conflict settings at the time of publishing that count as post-war today?

**>>> Author response:** Thank you for pointing this out. In line with the explanation above, studies about structural determinants of health inequalities in contexts other than post-conflict environments were excluded.

5) While I commend the authors for providing such extensive documentation to support their arguments, the large number of tables and overlap between them rather reduce the level of clarity.

The authors have provided helpful documentation (such as the PRISMA check list and flow chart and the data extraction sheet) and describe clearly how quality was assessed. But is it necessary to have both Table S8 that summarize the findings, and S1 as a data extraction sheet? Both have summaries of the results, but these are not identical. Similarly, is it necessary to have three separate tables (S2–S4) sorted by data quality, when this information is also available in the data extraction sheet?

**>>> Author response:** We thank the reviewer for the nice comments and suggestions. To increase the level of clarity, we have decreased the number of tables as follows:

We combined Tables S1 (Data Extraction Sheet) and S8 (Summary of study findings) into one table (New S1). Importantly, we moved the findings S8 to the new S1 Table to make sure the reported findings are identical. Finally, tables (S2–S4) have also been combined to cut down the number of tables.

I also find the ordering/names of the tables a bit peculiar. They are mentioned in the following order in the text: S10, S5, S6, S1, S7, S2, S3, S4, S8, S9. Perhaps they could be renamed according to the order in which they are presented? It would make the process and document audit trail more transparent.

**>>> Author response:** Thank you for your nice reminder. We revised the ordering and naming of the tables throughout the manuscript to make them clearer and have the numbering in line with the order in which they are mentioned in the manuscript.

6) “Specifically, little is known about the effects of conflict and displacement on the provision of Sexual and Reproductive Health (SRH) services [90]” on page 20. I do not agree with this statement. A substantial amount of literature has been published on this topic in Conflict and Health, BMJ Global Health, and other outlets. See also the RAISE initiative at Columbia University, the BRANCH Consortium, and intervention studies on the Minimum Initial Service Package (MISP).

**>>> Author response:** Thank you very much for your constructive feedback. We agree with you that more is currently known about the effects of conflict and displacement on the provision of Sexual and Reproductive Health (SRH) services in post-conflict settings. For the sake of clarity, we have rephrased this in the revised version of the manuscript: “Although a substantial amount of literature has been published on the effects of conflict and displacement on the provision of Sexual and Reproductive Health (SRH) services in post-conflict settings, there is a lot we do not know yet about how to address the problem. These findings concur with the conclusion of a recent systematic review of monitoring and evaluation indicators for SRH, showing that rigorous reporting on a core list of indicators is a prerequisite for identifying the changes that a country wants to see concerning SRH and the investments required to save lives [111].”

7) The section “Study identification” would be more logically placed before the section “Data extraction and analysis”, where the PRISMA chart is first mentioned. In fact, I do not see the point of having a separate heading for this section as it fits under “Study selection”.

**>>> Author response:** Thank you very much for your suggestion that helped us to improve this manuscript. This section has been revised accordingly.

8) It is interesting that, according to Table S7, Sub-Saharan Africa is substantially overrepresented in terms of geographical focus. Can this be reflected upon more directly? Does this reflect the incidence of post-conflict, or is it because of something else?

**>>> Author response:** Thank you very much for this great question. In our opinion, the fact that 27 out of 63 (42.8%) selected studies were conducted in Sub-Saharan Africa (as described on page 13) is an interesting finding on its own. Indeed, it could reflect a high incidence of post-conflict situations in this specific region. In their study on Violent Conflict and Its Impact on Health Indicators in sub- Saharan Africa, [David R. Davis](https://www.researchgate.net/scientific-contributions/David-R-Davis-2121746914?_sg%5B0%5D=vtUM_4wEUBkrE-8TITUoH6pj5mZfGmAcfE1PVsxN_0WEdLETIvk9oKwuBr6ohi1qSvfm1X8.al_l250z3h01lqRUKTtji0Bh6ZozhJ8GeKcuUR5592XcM0vkHV7DlEW5HZ6gq-E9SYJk0rhPGfwi_q3SGrZReQ.oOxa5Dm5qHNs5qo9ko4ShdQfaKQhpSmQHk7Iwj43kjokvYqwzncBE5iXZhmPmxMxNnVmYzTR9GqEFHfWiZNk_A&_sg%5B1%5D=vYIl_nusCMMvo6DRUNMeh4VKfxvzkqAci1OirFJW6jvOXl_5GIuDDhxfqvWrLBxh5wgLkuo.fCsn7KY8X20AC_4jL4ZvBy_dG14DbLAhPmWsuU_G89ZbuemBgxB6MiLBsDwcb7FgPjnJS_uE_uqrZYds2ZPJ2g) & [J. Kuritsky](https://www.researchgate.net/scientific-contributions/J-Kuritsky-2121716253?_sg%5B0%5D=vtUM_4wEUBkrE-8TITUoH6pj5mZfGmAcfE1PVsxN_0WEdLETIvk9oKwuBr6ohi1qSvfm1X8.al_l250z3h01lqRUKTtji0Bh6ZozhJ8GeKcuUR5592XcM0vkHV7DlEW5HZ6gq-E9SYJk0rhPGfwi_q3SGrZReQ.oOxa5Dm5qHNs5qo9ko4ShdQfaKQhpSmQHk7Iwj43kjokvYqwzncBE5iXZhmPmxMxNnVmYzTR9GqEFHfWiZNk_A&_sg%5B1%5D=vYIl_nusCMMvo6DRUNMeh4VKfxvzkqAci1OirFJW6jvOXl_5GIuDDhxfqvWrLBxh5wgLkuo.fCsn7KY8X20AC_4jL4ZvBy_dG14DbLAhPmWsuU_G89ZbuemBgxB6MiLBsDwcb7FgPjnJS_uE_uqrZYds2ZPJ2g) (2002) documented high levels of conflict in Sub-Saharan African countries but also poor health indicators. Furthermore, this could mean that Sub-Saharan Africa can be used to inform post-conflict planning when it involves determining the potential geographic extent strategy targeting health inequality-related questions.

9) A page number seems to be missing from the quote reference on page 14.

**>>> Author response:** Thank you very much for the comment. We have added a page number to help the reader immediately locate the source of the quote referenced on Page 14 from the original article.

**Reviewer #2:** Health inequality and or equity is an important area of studies while studying the same issue in the post-conflict setting has an added value in the literature. The authors did a fantastic job to compile all the important study findings and did a systematic review on the subject area. This has provided a solid basis for identifying the research gap and future research in this area. The study findings and the conclusion is consistent and very strong in terms of the claim. However, due to my limitation on the study methodology I am a bit concerned about the authors' strong claim of non applicability of the meta analysis due to the heterogeneity of the study settings, populations and interventions. Someone from the relevant field should be approached for her/his opinion on this matter. Finally, I am wondering whether the authors came across this paper "Improving Maternal Health Care in a Post Conflict Setting: Evidence from Chittagong Hill Tracts of Bangladesh, The Journal of Development Studies (2018); <https://doi.org/10.1080/00220388.2018.1554211> as it is not found in the reference or the list of studies covered.

**Response to Reviewer #2**

**>>> Author response**: We thank the reviewer for the positive feedback on our manuscript and for the suggestion on how we could further improve it. Although we understand the reviewer’s question about the methodological choice for not using meta-analysis in this review, we want to ensure the reviewer that we explored this aspect more in detail. As reported in the manuscript (Pg 9), before making the final decision on how to proceed “. …we contacted experts for advice (e.g., the plan to use PRISMA standard in combination with PRISMA-E was discussed with the editor in chief of the Campbell Collaboration Library)”.

For several reasons, we strongly believe that using qualitative approaches was appropriate to report on this systematic review:

- The approach allowed to describe, analyze, criticize, and summarize data by the vulnerable sub-groups,
- The qualitative inferences were presented as systematically as possible as part of the evidence in this systematic review.
- The study strengths and limitation section has a section explaining this as a limitation and more importantly how the authors have dealt with it.

Our choice to focus on the qualitative approaches was the most appropriate because the data available is so heterogeneous that a meta-analysis is impossible. If we would run the study again, we still would not choose a meta-analysis because of this heterogeneity. More importantly, we extracted the available data judiciously and purposively with an eye to what is relevant for our main review questions.

We finally thank the reviewer for helping us with important references: "Improving Maternal Health Care in a Post Conflict Setting: Evidence from Chittagong Hill Tracts of Bangladesh, The Journal of Development Studies (2018); <https://doi.org/10.1080/00220388.2018.1554211>

Although this important study was published during the timing of our data extraction, it was not included in our final selection for this review because the main focus of this study is not about inequality, the study evaluates a development program with an important maternal health care component in the Chittagong Hill Tracts of Bangladesh - a post-conflict setting.

We are currently working on the next manuscript on health inequality trends with a specific focus on Maternal Health Care – we will use this important reference to address any relevant questions.

**Reviewer #3: SUMMARY OF REVIEWER**

This review collates the current evidence on the structural determinants of health inequalities in post-conflict settings and describes the approaches used to address them. The methods and results were clearly described; however, the introduction and discussion should be re-structured to strengthen the study (and set up the importance/rationale of this review). Overall, this study will add important findings to the literature base on structural determinants of health in post-conflict settings.

INTRODUCTION

The authors have done a good job assessing the current evidence around health inequalities and conflict. However, the structure of the introduction felt slightly unclear and hard to follow. Sub-titles would help the reader follow the background narrative leading up to the rationale of the current study. Currently, the structure seems to be: inequalities persist globally and worsen in conflict; social determinants of health (SDH) in conflict; conflict in children; definition of inequalities vs inequities; definition of post-conflict settings; conflict as driver of health inequalities (ex DRC); right to health; impact of conflict on health and health systems documented but not on SDH; and jump to structural determinants of health inequalities in post-conflict settings. Review these paragraphs and find a structure that leads the reader to the gaps in the evidence and rationale of your study. For example, how are these inequalities specific to post-conflict settings and why is there less evidence on this area specifically. Start broad and then narrow on to the rationale of your review.

>>> Author response: We thank the reviewer for the suggested modifications in the introduction section. To lead the reader to the gaps in the evidence and rationale of our study, we revised the introduction section (from pg.3-8) as follows:

1. Sub-titles were created to improve the structure:

- *Social determinants versus Structural determinants of health*
- *Health inequalities versus health inequities*
- *Conflict versus post-conflict settings*
- *Right to health*

2. Paragraphs were moved in the background narrative leading up to the rationale of the current study.
3. Unnecessary details are removed from this section.

Specific points:

• The authors use the term ‘emerging countries.’ This would need to be defined or just stick with the term LMIC.

**>>> Author response:** Thank you for your comment and suggestion. we have rephrased this in the revised version of the manuscript: “…the social determinants affecting children in crisis settings are the same as those affecting children in Low and Middle-Income countries….”

• The authors define inequalities vs inequities in health (page 3). The definition of social determinants of health, specifically structural determinants of health, is needed higher up in the introduction and its definition should be referenced. Social determinants of health and structural determinants of health inequalities seem to be used interchangeably. Define and stick to the one which you will use for the review.

**>>> Author response:** Thank you very much for your constructive feedback. We have addressed your comments as described below:

“Social determinants versus Structural determinants of health

Because the concept of ‘social determinants’ refers simultaneously to both the determinants of health and the determinants of inequalities in health, we will expand our review of social determinants of health to include the terms ‘‘structural determinants of health’’ as we assess the literature on this topic. In this article, the term "structural determinants of health inequalities" refers specifically to the interplay between the socio-economic-political context, structural mechanisms generating social stratification, and the resulting socio-economic position of individuals. These mechanisms configure social groups' health opportunities based on their placement within hierarchies of power, prestige, and access to resources [9]. We hypothesize that structural determinants affect the distribution of resources and have the potential to influence mental health inequalities.”

• The outcome of interest (structural health inequalities) listed in methods needs to be defined as well (or instead of other combinations of those words).

**>>> Author response:** Thank you very much for these comments. We agree with the reviewer that the outcome of interest (structural health inequalities) should be defined as this is required to interpret the results. As noted above, the term "structural determinants of health inequalities" refers specifically to the interplay between the socio-economic-political context, structural mechanisms generating social stratification, and the resulting socio-economic position of individuals. These mechanisms configure social groups' health opportunities based on their placement within hierarchies of power, prestige, and access to resources [9]. We hypothesize that structural determinants affect the distribution of resources and have the potential to influence mental health inequalities.

• There is a lot of literature within the post-conflict setting on health. This felt like a summary of this was missing in the introduction.

**>>> Author response:** Thank you for emphasizing the need to clarify that there is already a lot of literature available on health within the post-conflict setting. As suggested, we have summarized this in the introduction (see page pg.3-8). Additionally, in reply to your main comment, we have restructured the introduction section to address your comments.

METHODS

The overall design of the study was appropriate and adequate to answer the research question. The search strategy, inclusion and exclusion criteria and study selection were adequately described.

Specific points:
• Add reference for why these vulnerable groups were selected (see comment later re general population).

**>>> Author response:** Thank you for this helpful suggestion. We have added references for why these vulnerable sub-groups were selected. To help the reader, we have slightly changed the text on page 7 of the manuscript as follows: “… the population of interest consisted of the most vulnerable population groups [34, 35] living in post-conflict areas such as the general population, women, children, internally displaced persons (IDPs), and the people with symptoms of mental illness – as these are generally affected the most.”, they were selected because, often, they have been referred to in the literature as most vulnerable in post-conflict environments.

• Upon reading the methods, I assumed you would organise the results according to the PROGRESS-Plus framework for structural determinants. Could this be further integrated into each section of the results? For example, within the children sub-section, describing the main PROGRESS-Plus risk factors.

**>>> Author response:** We thank the reviewer for this valid comment and the suggestions. The reviewer is right, it could have been possible (an option) to analyze our data using the PROGRESS-Plus framework for structural determinants on health inequalities in post-conflict settings. However, we initially discussed the way they would use this framework to guide/support this review. In the manuscript (Pg 8), we referred to the framework under search strategy, underlining the fact that it enables the inclusion of a range of factors needed to measure inequities. According to Jennifer O’Neill et al. (2013), the PROGRESS-PLUS acronym can be used as an aide-mémoire, a framework to guide data extraction. We used all PROGRESS-PLUS acronyms to make the best selection of the studies to be included in systematic reviews. To ensure that decisions about structural determinants of health inequalities should be targeted with any future intervention, we found it useful to involve the most vulnerable groups – therefore, the results have been organized according to the representatives from these sub-groups.

Although there could be some differences in the way these results were presented (PROGRESS-PLUS framework *versus* the most vulnerable sub-groups), these results depend entirely on how the studies were selected and if the quality assessment was done. In both situations, we believe the results are useful as they contribute to the body of knowledge on structural determinants of health inequalities in post-conflict settings.

RESULTS

The results answer the research question and are well presented.

Specific points:
• It would be helpful to the reader to add percentages (n/N total publications; X%) within the results section. For example, in the sentence “The majority of the studies were based on data collected in sub-Saharan Africa (n/N; X%), followed by South Asia and the Middle East and North Africa region.”

**>>> Author response:** Thank you very much for the helpful suggestions. We have integrated them in the results section (pg.13) as follows: “The majority of the studies were based on data collected in sub-Saharan Africa (27/63 total publications; 42%), followed by South Asia (7/63 total publications; 11%) and the Middle East (6/63 total publications; 9%) and North Africa region (6/63 total publications; 9%). Three regions, including Asia Pacific, South America, and Europe, had similar data - This is potentially an indicator that some recent peace agreements have been reached in these regions. Only one study collected data in the Russia and Eurasia region.”

• The general population was added as a vulnerable population group in the results but was not described in the methods. This should be added or explained. Furthermore, in this section, post-conflict was not mentioned. All results should reflect the specificities and complexities present in a post-conflict setting.

**>>> Author response:** Thank you for the helpful suggestions. We have integrated them in the methods section (pg.7) as follows: “The outcome of interest was structural health inequalities and the population of interest consisted of the most vulnerable population groups [35, 36] living in post-conflict areas such as the general population, women, children, IDPs, and people with symptoms of mental illness – as these are generally affected the most, they were selected because, often, they have been referred to in the literature as most vulnerable in post-conflict environments.” On several occasions, we have also made reference to post-conflict areas in the Materials and methods sections and sub-headings (from pages 7 to 12).

• IDP acronym is defined various times throughout the manuscript.

**>>> Author response:** Thank you very much for pointing this out. We revised this throughout the manuscript and used the IDP acronym appropriately.

• The term and section entitled – Mentally Ill. Could this be re-framed as people with symptoms of mental illness (see for reference of bias free language - <https://apastyle.apa.org/style-grammar-guidelines/bias-free-language/disability>).

**>>> Author response:** Thank you for your comments. As suggested by the reviewer, we have re-framed the term Mentally Ill as people with symptoms of mental illness throughout the manuscript.

DISCUSSION

An important strength of this work is its focus on post-conflict settings. The results should be reviewed to clearly state how the findings relate to the specific constraints/circumstances of a post-conflict setting, as at times it felt like unclear or not explicitly stated. For example, the sentence on children in armed conflict does not connect to the importance of this findings on a post-conflict setting.

Specific points:
• The paragraph about children is about armed conflict. Could this be better integrated into the evidence from your review about post-conflict?

**>>> Author response:** Thank you for your nice reminder. We revised the paragraph about children to make it clearer that it is more about post-conflict settings than conflict situations. The revised text reads as follows:

*“Children*

The effects of armed conflict on children’s physical health and development are not well understood. In particular, little is known about how the circumstances of a post-conflict setting could affect the health status of children. A recent systematic review of the effects of armed conflict on child health and development [8] shows some gaps in the medium and long-term effects. The authors report a striking paucity of data on child health in relation to conflict and further supports the need for research on how conflict and structural inequalities affect children and subsequent generations. According to the Human Security Centre, child mortality is high in post-conflict countries due to food insecurity leading to malnutrition and the disruption of the water supply and sanitation.[118]. This suggests that children are more vulnerable during and after conflicts, as a consequence of a quick transition from conflict to post-conflict in these environments”.

• Could sub-heading be added to the discussion? Again, this will help the reader follow the main findings. These could be based on sub-sections in the results section (population group and approaches to addressing health inequalities).

**>>> Author response:** Many thanks for the helpful suggestions. To help the reader follow the main findings of our review, we have integrated sub-headings in the discussion section of the manuscript (from pg.22-27) as follows: general population, Women, Children, IDPs, and Persons with symptoms of mental illness) and approaches to addressing health inequalities.

• A figure describing the recommendations for future research or summarising the gaps in the evidence base would be helpful to the readers.

>>> **Author response**: Although we are happy to add figures, we are not sure whether a visual representation would be the best way to present the recommendations and/or summarize the gaps. In fact, we are not even sure how such a figure would look like. If the reviewer has clear suggestions on this, then we are happy to reconsider.

 6. PLOS authors have the option to publish the peer review history of their article ([what does this mean?](https://journals.plos.org/plosone/s/editorial-and-peer-review-process#loc-peer-review-history)). If published, this will include your full peer review and any attached files.

**>>> Author response:** Thanks for your comment. We agree with you that publishing the peer review history increases transparency and accountability and helps to reinforce the validity of our research – it is good practice.

If you choose “no”, your identity will remain anonymous but your review may still be made public.

**Do you want your identity to be public for this peer review?** For information about this choice, including consent withdrawal, please see our [Privacy Policy](https://www.plos.org/privacy-policy).

Reviewer #1: No

Reviewer #2: **Yes:** Muhammad Badiuzzaman

Reviewer #3: No

[NOTE: If reviewer comments were submitted as an attachment file, they will be attached to this email and accessible via the submission site. Please log into your account, locate the manuscript record, and check for the action link "View Attachments". If this link does not appear, there are no attachment files.]

While revising your submission, please upload your figure files to the Preflight Analysis and Conversion Engine (PACE) digital diagnostic tool, <https://pacev2.apexcovantage.com/>. PACE helps ensure that figures meet PLOS requirements. To use PACE, you must first register as a user. Registration is free. Then, login and navigate to the UPLOAD tab, where you will find detailed instructions on how to use the tool. If you encounter any issues or have any questions when using PACE, please email PLOS at [figures@plos.org](mailto:figures@plos.org). Please note that Supporting Information files do not need this step.

*In compliance with data protection regulations, you may request that we remove your personal registration details at any time.* [*(Remove my information/details)*](https://www.editorialmanager.com/pone/login.asp?a=r)*. Please contact the publication office if you have any questions.*
