# Supplementary material for: Health inequalities in post-conflict settings: A systematic review
Source: PLoS One. 2022 Mar 14;17(3):e0265038. doi: 10.1371/journal.pone.0265038 (PMC8920275; doi:10.1371/journal.pone.0265038)
Supplement: Attachment — Submitted filename: Review sysrev plos one.docx [file pone.0265038.s011.docx]

Dear Editors,

Thank you for inviting me to review this manuscript a second time. I believe the authors have done a decent job addressing the comments by all reviewers in the first round of revision, but several major issues still remain before the manuscript is ready for publication.

* Why did you not do a database search to cover the period December 2020 and onwards? This would add to the longevity of the article, as it is already missing a whole year of potentially relevant publications for inclusion in the systematic review. Limiting the new search to the past 12 months should likely not be too time consuming.

* Even if the review is supposed to be restricted to post-conflict, there are some places where it sounds as if conflict is actually the main interest. The first 1.5 pages only covers conflict. The same goes for the paragraph “Many studies have investigated --- not well documented” on page 6. Another place is page 13 where the authors write “We found thirty studies --- health in conflict-affected general populations”. Why did you not write about post-conflict, if this is the context you are interested in?

* The introduction has some structural problems. It takes almost six pages before the authors explain what the article is about. I propose moving the paragraph “Very few empirical studies --- health inequalities in post-conflict settings” on pages 6-7 to the very beginning of the article.

* The subheadings “Social determinants vs structural determinants of health”, “Conflict versus post-conflict settings”, and “Right to health” are useful, but these sections would be more appropriately placed in a section called, for example, “Key concepts” or “Background”. This section should come after the introduction, where the authors should explain what they are about to do.

* There should be a new subheading after “Right to health” when the authors go back to explaining their study (the sentence starting with “Many studies have investigated…” on page 6).

* The paragraph “The outcome of interest was --- better paying job [35]” on page 7 should probably be cued earlier, so the reader knows already in the beginning that this is a key focus for the article.

* I am not convinced by the authors’ arguments to exclude LGBT+ as a vulnerable group. The Progress-Plus acronym includes Gender/Sex. It seems very peculiar to apply this category only to mean women, and not gender identity and sexual orientation.

* The review should not include the reference to Alzate (2008). The study selection criteria on page 9 says “a) post-conflict environment was explicitly described as study setting”. In the early 2000s, Colombia had the highest levels of conflict-related mortality. On page 17, the authors write: “From a human rights perspective, [42] notes that IDPs have been denied the opportunity to participate in the development process or their engagement has been seriously compromised in Colombia.” Alzate’s article does not discuss IDPs in a post-conflict scenario (which by any standard definitions, would be after the Havana Peace Accords signed in 2016). The Peace Accords have instated many programs to address health needs of IDPs. This article is just too old to be relevant for the case of Colombia, if the authors are indeed selecting on post-conflict settings.

* Reference 95 on page 16 about prenatal exposure is not correct. In this article, Roseboom writes “In fact, epigenetic studies have shown that prenatal exposure to violence leaves lasting epigenetic marks on the glucocorticoid receptor that can still be detected 10–19 years later.” Why do you refer to a literature review, and not the empirical article? Also, the wording of the sentence is a clear case of copy-paste. (The authors write: “Similarly, [95] notes that prenatal exposure to violence leaves lasting epigenetic marks on the glucocorticoid receptor that can still be detected 10–19 years later.”) I strongly encourage the authors to use their own words, and not refer to literature reviews but directly to empirical articles.

* On page 21 and many other places in the article, the authors refer to the general population as a population sub-group. A general population cannot also be a population sub-group. Please rephrase.

* The discussion seems quite repetitive/similar to the results. I suggest the authors to merge the discussion section until mid-page 26 into the results, and to let the parts starting with the “Study Strenghts and Limitations” be the discussion.

* In reference to the authors’ rebuttal with respect to IDPs versus refugees, I want to point out that not all international refugees enjoy protection from the UNHCR.

* On page 12, the authors write “Three regions, including Asia Pacific, South America, and Europe, had comparable data …”. What does “comparable data” mean? Please clarify.
