# Supplementary material for: Health inequalities in post-conflict settings: A systematic review
Source: PLoS One. 2022 Mar 14;17(3):e0265038. doi: 10.1371/journal.pone.0265038 (PMC8920275; doi:10.1371/journal.pone.0265038)
Supplement: Attachment — Submitted filename: Response to Reviewers.docx [file pone.0265038.s012.docx]

**Rebuttal letter**

**Response to Reviewer(s)**

PONE-D-21-08382R1

**Health inequalities in post-conflict settings: A systematic review.**

PLOS ONE

Dear Editor,

We would like to thank you and the reviewers for evaluating our manuscript and providing additional constructive comments. We appreciate your input and we have tried to address your suggestions about the reviewer’s comment as best as we could. We have copied the review decision from the submission menu of the editorial manager and used this format to write our responses. Below we provide the point-by-point responses to the comments. We carefully rechecked the entire manuscript for wordings that were not original and reworded them to represent original contributions. All modifications in the revised manuscript have been highlighted (with Track Changes). Of course, we remain at your disposal should you have any further suggestions for improvements to our paper.

Dear Editors,

Thank you for inviting me to review this manuscript a second time. I believe the authors have done a decent job addressing the comments by all reviewers in the first round of revision, but several major issues still remain before the manuscript is ready for publication.

* Why did you not do a database search to cover the period December 2020 and onwards? This would add to the longevity of the article, as it is already missing a whole year of potentially relevant publications for inclusion in the systematic review. Limiting the new search to the past 12 months should likely not be too time consuming.

>>> **Author Response:** Thank you for this very important insight into the design and conduct of this review. As explained in the search strategy (Page 7), the database search was a comprehensive search initiated in March 2018 and ultimately updated in December 2020. Thereafter, all selected papers were reviewed and our initial submission to PLoS One took place in March 2021. Hence, the year is mostly caused by the review time. That being said, we consulted PLOS ONE additional guidance (Sagoo GS et al., March 3, 2009) to critically look at the methodology. Not more than two years have passed between the searches and submission. However, we would be happy to update the database search if the reviewer would strongly feel the need for it, but this would require an additional extension.

* Even if the review is supposed to be restricted to post-conflict, there are some places where it sounds as if conflict is actually the main interest. The first 1.5 pages only covers conflict. The same goes for the paragraph “Many studies have investigated --- not well documented” on page 6. Another place is page 13 where the authors write “We found thirty studies --- health in conflict-affected general populations”. Why did you not write about post-conflict, if this is the context you are interested in?

>>> **Author Response:** Thank you for emphasizing the need to clarify the context we are interested in. Because the end of a conflict and the start of the post-conflict phase is difficult to determine, we have previously elaborated on what we understand under post-conflict settings. As explained on page 6 of our manuscript, post-conflict describes the **period immediately after a conflict is over.** But, the term is more difficult to define for many reasons. Firstly, conflicts do not necessarily end with the signing of official peace agreements. Secondly, low-intensity conflict replaces violent conflict because one or several actors are either excluded or not content with the peace agreement. The end of a conflict and subsequently the start of the post-conflict phase, therefore, become difficult to determine.

At this point, we still want to confirm that we are interested in a post-conflict setting for many reasons:

- this is indicated in the title of our manuscript: “ Health inequalities in post-conflict settings: A systematic review” – Page 1
- this systematic review aims to collate the current knowledge and identify knowledge gaps about structural determinants of health inequalities within post-conflict settings and assess the effects of approaches aimed at addressing structural determinants of health inequalities in this environment- Page 6
- the search strategy was comprehensive to allow for a wide variety of study designs and interpretations of structural determinants of health inequalities in post-conflict environments to be included – Pages 6-7

However, we clearly understood that additional clarifications were needed to orient the reader in a post-conflict-focused systematic review. By explicitly referring to conflict and post-conflict environments instead of conflict only, we rephrased the following in the manuscript:

- Conflict and post-conflict environments are now referred to in the first 1.5 pages instead of conflict only.
- On page 6: The same goes for the paragraph “Many studies have investigated --- not well documented”.
- As regards the term ‘conflict-affected’: we use this term because, obviously, the interest in post-conflict areas arises from the very fact that the conflict-affected the health situation of the population.
- On page 14: the text reads as follows “We found thirty studies --- health in conflict and post-conflict environments”.

* The introduction has some structural problems. It takes almost six pages before the authors explain what the article is about. I propose moving the paragraph “Very few empirical studies --- health inequalities in post-conflict settings” on pages 6-7 to the very beginning of the article.

>>> **Author Response:** Thank you very much for your feedback! As the reviewer suggested, we have moved the section starting with “Very few empirical studies --- health inequalities in post-conflict settings” on pages 6-7 to the very beginning of the article. We added the following paragraph at the first page of the Introduction:

” Very few empirical studies have measured health equity in conflict-affected settings, but those have suggested that conflict has a significant impact [4]. Most importantly, there is limited evidence on appropriate approaches for health equity in such environments. To accomplish equity, the prerequisite is first to ascertain the presence of inequality so that targeted interventions can be planned, implemented, and strategically monitored to determine the progress [5]. This systematic review aims to collate the current knowledge and identify knowledge gaps about structural determinants of health inequalities within post-conflict settings and assess the effects of approaches aimed at addressing structural determinants of health inequalities in this environment. It contributes to the body of knowledge on structural determinants of health inequalities in post-conflict settings which is also the main outcome of this study”.

We hope this helps to immediately indicate to the reader what the article is about.

* The subheadings “Social determinants vs structural determinants of health”, “Conflict versus post-conflict settings”, and “Right to health” are useful, but these sections would be more appropriately placed in a section called, for example, “Key concepts” or “Background”. This section should come after the introduction, where the authors should explain what they are about to do.

>>> **Author Response:** Thank you for your comment. We followed the instructions provided by the reviewer and created a new section after the introduction. This section is named: Key concepts. To explain to the reader what we main by key concepts, we added the following paragraph at the beginning of this new section as follows:

” To study health inequalities, it is of importance to clarify the following concepts: social determinants *versus* structural determinants of health; inequalities *versus* inequities in health; conflict *versus* post-conflict settings, effects of conflict on health equity and right to health is relevant. Without clearly understanding those key concepts, no one can develop effective policies for health equity. This paper helps to define those key questions that are needed to better understand health inequalities in post-conflict settings, such as...”

* There should be a new subheading after “Right to health” when the authors go back to explaining their study (the sentence starting with “Many studies have investigated…” on page 6).

>>> **Author Response:** Thank you very much for your suggestion that helped us to improve the structure of the introduction section. We added a new subheading after ”Right to health” This section has been named: ”Effects of conflict on health equity*”-* see page 7.

* The paragraph “The outcome of interest was --- better paying job [35]” on page 7 should probably be cued earlier, so the reader knows already in the beginning that this is a key focus for the article.

>>> **Author Response:** Thank you very much for pointing this out. We tend to agree with the reviewer that moving the section referred to in the comment, might help the reader to capture the key focus for the article from the beginning. We have carefully tried to cue this section earlier. Even so, we feel it still fits better where it is right now because the other paragraphs from the introduction focus more on study rationale, literature review, and key concepts - see page 7. To help the reader to capture the key focus for the article from the beginning, we rephrased the following on page 3 - specifically referring to the main study outcome as follows:

”It contributes to the body of knowledge on structural determinants of health inequalities in post-conflict settings which is also the main outcome of this study”.

* I am not convinced by the authors’ arguments to exclude LGBT+ as a vulnerable group. The Progress-Plus acronym includes Gender/Sex. It seems very peculiar to apply this category only to mean women, and not gender identity and sexual orientation.

>>> **Author Response:** Thank you very much for coming back to this relevant question. In reality, many people continue to face acute violence and discrimination based on their sexual orientation and gender identity (OHCHR, 2011), and this violence can be exacerbated in post-conflict times. The very complex post-conflict environment is characterized by large numbers of vulnerable people to be absorbed into civilian life. Additionally, there is a reduced functioning of coping mechanisms at the individual, community, and national levels and many other demands to be met. For these reasons and many others (such as the institutional destruction to be addressed), we very much agree with the reviewer’s assessment that LGBTQ+ people belong to the vulnerable population sub-groups in post-conflict environments. However, it might be more relevant to place this into the right perspective by defining what is meant by post-conflict vulnerability, who is vulnerable, and to what particular risk?

How gender identity and sexual orientation (about inequality in health) should be handled in a post-conflict context is indeed an important issue. As noted previously, the focus of our review was to collate the current knowledge and knowledge gaps about structural determinants of health inequalities within post-conflict settings and assess the effects of approaches aimed at addressing structural determinants of health inequalities in this environment. In particular, we found sufficient empirical evidence on gender identity in our review, but less on sexual orientation concerning inequality in health, suggesting that this important issue cannot be easily summarized in the current manuscript. Luckily, we can already learn about this topic from a stable (non-conflict) context. Findings from a recent comprehensive UK scoping review about the Politics of LGBT+ Health Inequality, Elizabeth McDermott et al. (2021) notes that the current body of UK LGBT+ health inequality research is relatively small but there is clear evidence of health inequities between the LGBT+ community, suggesting that the evidence base is insufficient to address this nationally recognized health inequality. Thus, although many people agree (and we are among them) and recognize that LGBTQ+ people as part of the most vulnerable populations (particularly in post-conflict settings) - they require special attention when developing public health interventions that can successfully tackle LGBT+ health inequality. Special consideration must be given to the particular needs and risks faced by individuals based on their sexual orientation and gender identity (e.g. LGBTQ+ people).

Because the reviewer is a strong proponent of including LGBTQ+ people as a separate sub-group in our review, we would not feel comfortable discussing this sub-group without giving it proper attention in the search terms used to identify publications. Thus, this would simply require running an entirely new search and writing a new paper on this (which we may consider doing in the future given the socially relevant topic). For now, we prefer to point the reader in the direction of the sub-groups we have identified as being most vulnerable in this environment.

* The review should not include the reference to Alzate (2008). The study selection criteria on page 9 say “a) post-conflict environment was explicitly described as study setting”. In the early 2000s, Colombia had the highest levels of conflict-related mortality. On page 17, the authors write: “From a human rights perspective, [42] notes that IDPs have been denied the opportunity to participate in the development process or their engagement has been seriously compromised in Colombia.” Alzate’s article does not discuss IDPs in a post-conflict scenario (which by any standard definitions, would be after the Havana Peace Accords signed in 2016). The Peace Accords have instated many programs to address health needs of IDPs. This article is just too old to be relevant for the case of Colombia, if the authors are indeed selecting on post-conflict settings.

>>> **Author Response:** We thank the reviewer for this valid comment and the suggestions. Although the paper by Alzate was initially included because it fulfilled the search criteria, we removed it. Furthermore, we still want to emphasize that on page 6 of our manuscript, we referred to additional criteria for the final selection of publications including - *(i) containing at least one of the three key concepts (post-war, inequality, and health) (S2 File).*

Indeed, the reviewer is right by pointing out that Colombia shouldn’t be seen as a post-conflict setting by the time Alzate’s article was published in 2008. One argument supporting this statement is that we didn’t find a signed peace agreement (part of the characteristics used to define post-conflict settings in our manuscript) this time.

To better understand the history of the conflict in Colombia, we checked and found the classification by Ramos Jaraba et al. (2020) relevant to this discussion - see summary below:

| **Period/years** | **Description** |
| --- | --- |
| **FIRST PERIOD (1958–1982)** | Emergence of guerrilla forces, subversive violence, and violence between two political parties. |
| **SECOND PERIOD (1982–1996)** | Was defined by the military strengthening of guerrilla forces and their territorial expansion, as well as the appearance of paramilitaries and proliferation of drug trafficking. Also there were peace process with some guerrilla groups. |
| **THIRD PERIOD (1996–2005)** | Exhibited the greatest upsurge in the conflict with the expansion of illegal armed groups, massacres, extortion, kidnapping, drug trafficking. |
| **FOURTH PERIOD (2005–2012)** | Known as the period of “readjustment of the armed conflict,” was defined by two crucial processes: 1) a military offensive by the Colombian state, and 2) negotiations and subsequent demobilization of paramilitary groups. |
| **FIFTH PERIOD (2012–2017)** | Peace dialogues in Havana between the national government and FARC-EP guerillas, dialogue with ELN guerillas in Quito, reconfiguration of other armed groups in the country. |

Alzate’s paper was published in the fourth period of the long conflict. Although this period does not refer to a signed peace agreement in Colombia, it indicates that negotiations and demobilization of paramilitary groups were ongoing.

Finally, we addressed all comments and removed the reference to Alzate (2008) through the manuscript. We also updated supplemental study materials (PRISMA and extraction table) to reflect this change.

* Reference 95 on page 16 about prenatal exposure is not correct. In this article, Roseboom writes “In fact, epigenetic studies have shown that prenatal exposure to violence leaves lasting epigenetic marks on the glucocorticoid receptor that can still be detected 10–19 years later.” Why do you refer to a literature review, and not the empirical article? Also, the wording of the sentence is a clear case of copy-paste. (The authors write: “Similarly, [95] notes that prenatal exposure to violence leaves lasting epigenetic marks on the glucocorticoid receptor that can still be detected 10–19 years later.”) I strongly encourage the authors to use their own words, and not refer to literature reviews but directly to empirical articles.

>>> **Author Response:** Thank you very much for your constructive feedback. We have addressed your comments as described below.

Page 19. In a study of the effects of the conflict on mortality both in utero and during the first year of life in the DRC, Dagnelie and colleagues [64] show that in utero exposure to conflict negatively affects the number of males (by a reduction of the number of male fetuses) reaching birth. Furthermore, the authors show that conflict exposure increases infant mortality specifically among girls. Possible explanations for such an outcome are in utero biological factors.

In another study discussing why achieving gender equality is of fundamental importance to improve the health and well-being of future generations, Roseboom (94) notes that prenatal exposure to violence has been associated with long-term effects on children’s health, especially on brain structure and neural function, that can still be detected many years later.

* On page 21 and many other places in the article, the authors refer to the general population as a population sub-group. A general population cannot also be a population sub-group. Please rephrase.

>>> **Author Response:** Thank you for constructive feedback. For clarification, this has now been rephrased and changed to “civilian population ”. Accordingly, it has also been modified throughout the revised manuscript.

* The discussion seems quite repetitive/similar to the results. I suggest the authors to merge the discussion section until mid-page 26 into the results, and to let the parts starting with the “Study Strenghts and Limitations” be the discussion.

>>> **Author Response:** We understood that clarifications were needed to avoid repetitions in the results section. As suggested by the reviewer, we merged the discussion section until mid-page 26 into the results. As a result, the parts starting with the “Study Strengths and Limitations” are now in the discussion section.

* In reference to the authors’ rebuttal with respect to IDPs versus refugees, I want to point out that not all international refugees enjoy protection from the UNHCR.

>>> **Author Response:** Thanks for your comment. We agree with the reviewer that not all international refugees enjoy protection from the UNHCR.

* On page 12, the authors write “Three regions, including Asia Pacific, South America, and Europe, had comparable data …”. What does “comparable data” mean? Please clarify.

>>> **Author Response:** Thank you very much for pointing this out. We rephrased the sentence to explain what we mean by comparable data. The revised text on page 13 reads as follows: ”

”Small numbers of papers emanated from three regions, including Asia Pacific, South America, and Europe – potentially an indicator that recent peace agreements have been reached in these regions. Only one study collected data in the Russia and Eurasia region.”
