# Supplementary material for: Health inequalities in post-conflict settings: A systematic review
Source: PLoS One. 2022 Mar 14;17(3):e0265038. doi: 10.1371/journal.pone.0265038 (PMC8920275; doi:10.1371/journal.pone.0265038)
Supplement: Attachment — Submitted filename: Response to Reviewers.docx [file pone.0265038.s013.docx]

**Rebuttal letter**

**Response to Reviewer(s)**

PONE-D-21-08382R2

**Re: Resubmission of manuscript “Health inequalities in post-conflict settings: A systematic review”.**

PLOS ONE

Dear Academic Editor,

We would like to thank you and the reviewers for the opportunity to revise and resubmit our manuscript to PLOS ONE. We further appreciate your input and we have addressed the remaining minor comments. We have copied the review decision from the submission menu of the editorial manager and used this format to write our responses. Below we provide the point-by-point responses to the comments raised. All modifications in the revised manuscript have been highlighted (with Track Changes). Of course, we remain at your disposal should you have any further suggestions for improvements to our paper.

Dear Dr. Bwirire,

Thank you for submitting your manuscript to PLOS ONE. After careful consideration, we feel that it has merit but does not fully meet PLOS ONE’s publication criteria as it currently stands. Therefore, we invite you to submit a revised version of the manuscript that addresses the points raised during the review process.

Please submit your revised manuscript by Mar 17 2022 11:59 PM. If you will need more time than this to complete your revisions, please reply to this message or contact the journal office at [plosone@plos.org](mailto:plosone@plos.org). When you're ready to submit your revision, log on to <https://www.editorialmanager.com/pone/> and select the 'Submissions Needing Revision' folder to locate your manuscript file.

Please include the following items when submitting your revised manuscript:

- A rebuttal letter that responds to each point raised by the academic editor and reviewer(s). You should upload this letter as a separate file labeled 'Response to Reviewers'.
- A marked-up copy of your manuscript that highlights changes made to the original version. You should upload this as a separate file labeled 'Revised Manuscript with Track Changes'.
- An unmarked version of your revised paper without tracked changes. You should upload this as a separate file labeled 'Manuscript'.

If you would like to make changes to your financial disclosure, please include your updated statement in your cover letter. Guidelines for resubmitting your figure files are available below the reviewer comments at the end of this letter.

If applicable, we recommend that you deposit your laboratory protocols in protocols.io to enhance the reproducibility of your results. Protocols.io assigns your protocol its own identifier (DOI) so that it can be cited independently in the future. For instructions see: [https://journals.plos.org/plosone/s/submission-guidelines#loc-laboratory-protocols](https://journals.plos.org/plosone/s/submission-guidelines#loc-laboratory-protocols). Additionally, PLOS ONE offers an option for publishing peer-reviewed Lab Protocol articles, which describe protocols hosted on protocols.io. Read more information on sharing protocols at <https://plos.org/protocols?utm_medium=editorial-email&utm_source=authorletters&utm_campaign=protocols>.

We look forward to receiving your revised manuscript.

Kind regards,

Jakob Pietschnig, PhD, MSc

Academic Editor

PLOS ONE

Journal Requirements:

Please review your reference list to ensure that it is complete and correct. If you have cited papers that have been retracted, please include the rationale for doing so in the manuscript text, or remove these references and replace them with relevant current references. Any changes to the reference list should be mentioned in the rebuttal letter that accompanies your revised manuscript. If you need to cite a retracted article, indicate the article’s retracted status in the References list and also include a citation and full reference for the retraction notice.

[Note: HTML markup is below. Please do not edit.]

**Reviewers' comments:**

**Reviewer's Responses to Questions**

**Comments to the Author**

1. If the authors have adequately addressed your comments raised in a previous round of review and you feel that this manuscript is now acceptable for publication, you may indicate that here to bypass the “Comments to the Author” section, enter your conflict of interest statement in the “Confidential to Editor” section, and submit your "Accept" recommendation.

Reviewer #1: All comments have been addressed

2. Is the manuscript technically sound, and do the data support the conclusions?

The manuscript must describe a technically sound piece of scientific research with data that supports the conclusions. Experiments must have been conducted rigorously, with appropriate controls, replication, and sample sizes. The conclusions must be drawn appropriately based on the data presented.

Reviewer #1: Yes

3. Has the statistical analysis been performed appropriately and rigorously?

Reviewer #1: N/A

4. Have the authors made all data underlying the findings in their manuscript fully available?

The [PLOS Data policy](http://www.plosone.org/static/policies.action#sharing) requires authors to make all data underlying the findings described in their manuscript fully available without restriction, with rare exception (please refer to the Data Availability Statement in the manuscript PDF file). The data should be provided as part of the manuscript or its supporting information, or deposited to a public repository. For example, in addition to summary statistics, the data points behind means, medians and variance measures should be available. If there are restrictions on publicly sharing data—e.g. participant privacy or use of data from a third party—those must be specified.

Reviewer #1: Yes

5. Is the manuscript presented in an intelligible fashion and written in standard English?

PLOS ONE does not copyedit accepted manuscripts, so the language in submitted articles must be clear, correct, and unambiguous. Any typographical or grammatical errors should be corrected at revision, so please note any specific errors here.

Reviewer #1: Yes

6. Review Comments to the Author

Please use the space provided to explain your answers to the questions above. You may also include additional comments for the author, including concerns about dual publication, research ethics, or publication ethics. (Please upload your review as an attachment if it exceeds 20,000 characters)

Reviewer #1:

This manuscript has been much improved. For example, I particularly like that the authors use the term “civilian population” rather than “general population” and explain their rationale for this.

Some minor comments remain before the manuscript is ready for publication:

**Reviewers' comment:**

On inclusion/exclusion of LGBT as a vulnerable group: the authors’ counterarguments are fine, but then I suggest adding this as a limitation and a gap for future work. Picking two or three sentences from the rebuttal letter would suffice.

**>>> Author Response:**

We thank the reviewer for this valid comment and the suggestions. The revised text on page 27 of our manuscript reads now as follows:

”Third, although we agree and recognize LGBTQ+ (Lesbian, Gay, Bisexual, Trans, Queer, and others) people as belonging to the most vulnerable sub-groups in post-conflict settings – this sub-group was not included in our systematic review because LGBTQ+ issues require special attention. For the same reason, they could not be easily summarized in the current manuscript. To successfully tackle LGBTQ+ issues, special consideration must be given to the particular needs and risks faced by individuals based on their sexual orientation and gender identity. Future systematic reviews on health inequalities within the post-conflict environment should also include LGBTQ+ people as a vulnerable sub-group”.

**Reviewers’ comment:**

The paragraph on page 4 starting with: ”There is evidence that conflict can be a primary driver of health inequalities (9). The following example from the DRC clearly illustrates this point.” What does this example have to do with health inequalities? It’s more about the difference between direct and indirect mortality in conflict. For it to have something to do with health inequalities, it would have needed information about how mortality differs across SES groups.

>>> **Author Response:**

We thank the reviewer for raising this question. We went back to the paper in which the relionship between conflict and health inequalities has been extensively discussed (Ranson et al, 2007). Specifically, we focused on the broad context of interest for our systematic review, which includes inequality in health within the post-conflict environment. While reading Ranson’s review again - a few arguments came up that explain why we selected the example from the DRC to illustrate the link between conflict and health inequalities (which we have made explicit in the revised version of the manuscript – as described below).

First, we agree with the reviewer that socio-economic status (as measured by education, income or occupation) is a key determinant of health. However, there are other relevant determinants of health which are specific to the post-conflict environment, such as: 1) differential exposure – which may vary (by type, amount, and duration) between social groups 2) differential vulnerability – even when a given risk factor is distributed evenly across social groups, its impact on health may be unevenly distributed due to underlying differences between social groups in their vulnerability or susceptibility to that factor and 3) differential consequences – the impact of a certain health event differs depending on an individual’s or family’s socio-economic circumstances or health.

Second, life expectancy and mortality trends are familiar ways of measuring health status and monitoring health inequalities. The example of the DRC illustrates the links between conflict and geographical inequalities in mortality, suggesting that mortality rate was higher in unstable eastern provinces, and supporting the idea that life expectancy is lower in more deprived areas.

To clearly explain this point to the reader, we have revised the paragraph on page 4 of the manuscript, emphasizing the link between conflict and health inequalities in the DRC, and we have rephrased this sentence as follows:

”There is evidence that conflict can be a primary driver of health inequalities (9). Specifically, socio-economic status (as measured by education, income or occupation) is a key determinant of health. But, there are other more important determinants of health which are specific to the post-conflict environment, such as : 1) differential exposure – which may vary (by type, amount, and duration) between social groups 2) differential vulnerability – even when a given risk factor is distributed evenly across social groups, its impact on health may be unevenly distributed due to underlying differences between social groups in their vulnerability or susceptibility to that factor and 3) differential consequences – the impact of a certain health event differs depending on an individual’s or family’s socio-economic circumstances or health.

Furthermore, life expectancy and mortality trends are familiar ways of measuring health status and monitoring health inequalities. Countries without basic data on mortality (including post-conflict countries), by socioeconomic indicators might have difficulties progressing their health equity agenda. The example of the DRC illustrates the links between conflict and regional inequalities in mortality. It indicates that mortality rate was higher in unstable eastern provinces compared to stable provinces, and suggest that life expectancy was lower in more deprived areas.

Through a series of mortality surveys conducted between 2000 and 2004, the International Rescue Committee (IRC) has documented the humanitarian impact of war and conflict in the DRC. These studies estimated that 3.9 million people had died since 1998, arguably making the DRC's conflict the world's deadliest crisis since World War II. Less than 10 percent of all deaths were due to violence, with most deaths attributed to easily preventable and treatable health conditions (indirectly caused by conflict- and resulting in inequitable health) such as malaria, diarrhea, respiratory infections, and malnutrition (10). Furthermore, deaths are often used as the primary indicator for the effect that conflict has on civilians; indeed, the number of these deaths declined in the DRC following an increase in security (11-13).”

**Reviewers' comment:**

Page 13 “… potentially an indicator that recent peace agreements have been reached in these regions.” This can easily be checked, so there is no need for speculation. See for example <https://www.peaceagreements.org/>. I would assume Sub-Saharan Africa is more researched due to the many international development cooperation initiatives in the region.

>>> **Author Response:**

Thank you for sharing this very interesting hyperlink about peace agreements status. We checked the Peace Agreement Database (<https://www.peaceagreements.org/>) and found relevant information about the specific regions of interest (e.g.: Asia Pacific, South America, and Europe). Between 1990 and 2021, 381 peace agreements were signed in the Asia Pacific, 414 in Europe, and 195 in the Americas - which support our previous reporting that recent peace agreements were reached in these regions. For the sake of clarity, we have rephrased this in the revised version of the manuscript on Page 13: “…an indicator that recent peace agreements have been reached in these regions ”, omitting the ‘potentially’.

**Reviewers' comment:**

Page 17 “According to Sorenson (115), one feature of post-conflict environments that is often ignored by academics and policymakers is the situation of women.” What does this mean? Women’s situation in post-conflict has been extensively researched.

>>> **Author Response:**

Thank you very much for your question. We agree with the reviewer that women’s situation in post-conflict has been extensively researched and findings suggest that their situation is characterized by fundamental weaknesses or imperfection in several ways (e.g. Targeting women for professional health care training is a key to improving the community's health status, In addition, it may also provide valuable opportunities for long-term employment. However, in practice, many programs continue to discriminate against women when enrolling people in training activities). This has serious implications for both our understanding of women's situation and our ability to effectively and efficiently assist them.

To explain what we mean by “often ignored”; we revised the sentence on page 17 as follows:

“According to Sorenson (115), health care and other social facilities remain inadequate for women’s health in the post-conflict period. As such, they face new challenges (e.g. their traditional roles and relationship) and inherit additional responsibilities. The social transformation occurring in the post-conflict context opens up opportunities that should not be missed for women ”.

**Reviewers' comment:**

References # 14 and # 105 (and possibly others) are not complete.

>>> **Author Response:**

Thank you very much for the reminder. For completeness, missing details were added to references # 14 and #105 in EndNote. Furthermore, relevant details were added to references #7, #10, #13, #16, #18, #20, #21, #22, #23, #28, #32, #37, #59 and #105 in the revised manuscript.

**Reviewers' comment:**

Last sentence on page 5: “throughout” – not “thought”.

>>> **Author Response:**

Thank you for this comment. We have made revisions accordingly. The original sentence on page 5 of the manuscript has been modified with the inclusion of the correct word: ”throughout”. As a result we believe the message now is more clear.

7. PLOS authors have the option to publish the peer review history of their article ([what does this mean?](https://journals.plos.org/plosone/s/editorial-and-peer-review-process#loc-peer-review-history)). If published, this will include your full peer review and any attached files.

If you choose “no”, your identity will remain anonymous but your review may still be made public.

**Do you want your identity to be public for this peer review?** For information about this choice, including consent withdrawal, please see our [Privacy Policy](https://www.plos.org/privacy-policy).

Reviewer #1: No

[NOTE: If reviewer comments were submitted as an attachment file, they will be attached to this email and accessible via the submission site. Please log into your account, locate the manuscript record, and check for the action link "View Attachments". If this link does not appear, there are no attachment files.]

While revising your submission, please upload your figure files to the Preflight Analysis and Conversion Engine (PACE) digital diagnostic tool, <https://pacev2.apexcovantage.com/>. PACE helps ensure that figures meet PLOS requirements. To use PACE, you must first register as a user. Registration is free. Then, login and navigate to the UPLOAD tab, where you will find detailed instructions on how to use the tool. If you encounter any issues or have any questions when using PACE, please email PLOS at [figures@plos.org](mailto:figures@plos.org). Please note that Supporting Information files do not need this step.

*In compliance with data protection regulations, you may request that we remove your personal registration details at any time.* [*(Remove my information/details)*](https://www.editorialmanager.com/pone/login.asp?a=r)*. Please contact the publication office if you have any questions.*
